# Chemically modified nucleic acids and DNA intercalators as tools for nanoparticle assembly

**DOI:** 10.1039/d1cs00632k

**Published:** 2021-11-18

**Authors:** Angela F. De Fazio, Doxi Misatziou, Ysobel R. Baker, Otto L. Muskens, Tom Brown, Antonios G. Kanaras

**Affiliations:** School of Physics and Astronomy, Faculty of Engineering and Physical Sciences, University of Southampton Southampton SO17 1BJ UK A.Kanaras@soton.ac.uk; Istituto Italiano di Tecnologia, Via Morego 30 16163 Genova Italy; Department of Chemistry, University of Oxford, Chemistry Research Laboratory 12 Mansfield Road Oxford OX1 3TA UK; Institute for Life Sciences, University of Southampton Southampton SO17 1BJ UK

## Abstract

The self-assembly of inorganic nanoparticles to larger structures is of great research interest as it allows the fabrication of novel materials with collective properties correlated to the nanoparticles’ individual characteristics. Recently developed methods for controlling nanoparticle organisation have enabled the fabrication of a range of new materials. Amongst these, the assembly of nanoparticles using DNA has attracted significant attention due to the highly selective recognition between complementary DNA strands, DNA nanostructure versatility, and ease of DNA chemical modification. In this review we discuss the application of various chemical DNA modifications and molecular intercalators as tools for the manipulation of DNA-nanoparticle structures. In detail, we discuss how DNA modifications and small molecule intercalators have been employed in the chemical and photochemical DNA ligation in nanostructures; DNA rotaxanes and catenanes associated with reconfigurable nanoparticle assemblies; and DNA backbone modifications including locked nucleic acids, peptide nucleic acids and borane nucleic acids, which affect the stability of nanostructures in complex environments. We conclude by highlighting the importance of maximising the synergy between the communities of DNA chemistry and nanoparticle self-assembly with the aim to enrich the library of tools available for the manipulation of nanostructures.

## Abstract 2

The chemical manipulation of DNA offers new tools to tune the properties of nanoparticle self assemblies.

## Graphical abstract

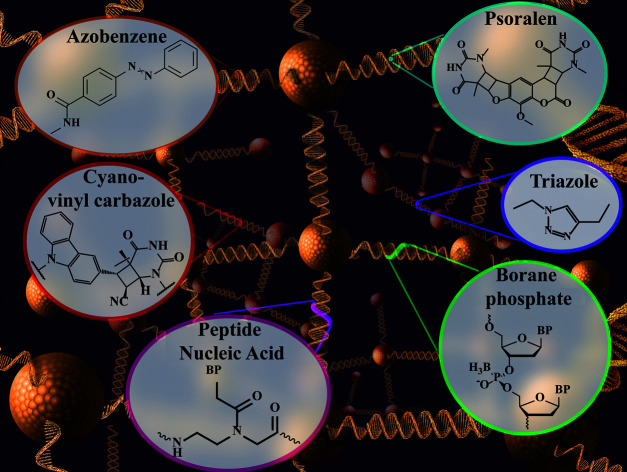

## 1. Introduction

There have been significant advancements in controlling the nanoscale features of materials with the aim of fabricating materials with desired properties.^1^ The methods employed, to achieve this goal, have been based on top-down or bottom-up approaches. A typical example of a top-down approach is the use of an external source (*e.g.* an electron beam) to design nanoscale features on a bulk surface while a bottom-up approach relies on the organisation of building units (*e.g.* molecules, atoms, nanoparticles) to form larger structures.^2–5^ The main difference between these two methods, is that the instructions for the design of the material in the top-down approaches are provided externally, whilst in the bottom-up methods the design information is encoded within the building units.^5–7^ Whilst a top-down approach to make materials is often simpler to execute but usually more expensive, a bottom up-approach can offer a broader versatility of structures due to the availability of more methods to chemically encode design rules in the building units.^6^

Employing inorganic nanoparticles as building units to fabricate larger structures is of particular interest owing to their physicochemical properties, which are significantly different from their bulk counterparts. These novel properties arise from their small size and large surface-to-volume ratio, which can be tuned by changing their chemical composition, size, and shape.^8–13^ Recent advances in nanoparticle synthesis have made available a large library of nanoparticles of various morphologies. Metallic, metal oxide, semiconductor, magnetic, upconversion and perovskite nanoparticles have been developed in a variety of sizes and shapes,^14–21^ and many of these can be prepared on a large scale.^22–25^ The surface chemistry of these nanomaterials has been significantly explored and a versatile range of ligands have been reported to enable the stability of nanoparticles in complex environments and enrich them with appropriate functionalities for a diverse range of applications.^26–30^

Whilst many types of ligands have been developed to direct the assembly of nanoparticles,^31–40^ DNA has attracted significant interest.^41–49^ DNA has several advantages over other ligands, which include: (i) a DNA strand binds selectively and specifically to its complementary sequence, (ii) a plethora of DNA structures can be made using simple methods, (iii) DNA can interact with other functional molecules (*e.g.* enzymes) in a specific manner, and (iv) DNA strands can be chemically modified to include functional chemical groups. The functionalisation of nanoparticles with DNA and the ability of DNA to guide the self-assembly of nanoparticles were first reported by the groups of Alivisatos and Mirkin in 1996.^50,51^ In their study, Mirkin and co-workers functionalised the surface of gold nanoparticles with a dense layer of thiol-modified oligonucleotides. By mixing two batches of DNA functionalised nanoparticles with a complementary DNA target, a macroscopic amorphous aggregate was obtained.^50^ In an alternative approach, Alivisatos and collaborators attached a discrete number of DNA strands to direct the assembly of gold nanoparticles into small oligomers of particles.^47,52–54^

Both groups demonstrated the powerful concept that DNA-coated nanoparticles can be considered as “artificial atoms”, which can assemble into a versatile range of larger structures with novel properties. These pioneering studies led to the rapid development of the field of material engineering with DNA.^46,55,56^ In 2008, groups led by Mirkin and Gang independently reported the formation of ordered three dimensional (3D) DNA gold nanoparticle superlattices with face-centred cubic and body-centred cubic configurations.^57,58^ Since then, a vast range of nanoparticle crystals have been fabricated.^59–65^ Particles with different chemical compositions have been organised into homo- and heterogeneous crystal structures. For example, silver nanoparticles and binary silver–gold lattices,^66^ cadmium selenide quantum dots and gold nanoparticle binary lattices,^67^ and mixed gold (Au) and upconversion (UC) nanoparticles assemblies^68^ have been reported.

DNA design has also been explored to make a broader range of nanoparticle assemblies. Examples include the design of engineered DNA origami frames to arrange gold nanoparticles in specific lattice configurations,^69,70^ the fabrication of crystalline structures with systematically positioned defects,^71^ the dynamic re-arrangements of nanoparticles utilising reconfigurable DNA strands,^72^ competitive displacement,^73^ and electrostatic interactions.^74–76^ For example, complex origami polyhedra have been employed as valence-controlled building blocks, named DNA material voxels.^77^ A voxel is a three-dimensional pixel, taking into account the volume component to create a larger three-dimensional picture. Analogously, a DNA material voxel is a three-dimensional DNA polyhedron with prescribed valence that integrates a nano-object within the scaffold. Each voxel represents a defined building unit of 3D space, which can be empty or occupied; the valence and coordination of each individual voxel is determined by the frame's geometry (*e.g.* tetrahedral, octahedral or cubic), which can bind to each other through hybridisation. This allows the definition of a lattice symmetry and a lattice composition through the material voxel design and enables the rational assembly of 3D ordered nanomaterials from desired nano-objects for a broad range of applications.^78,79^

Moreover, molecular recognition has been employed as a tool to guide DNA-nanoparticle self-assembly. The introduction of specific DNA sequences can encode for the molecular recognition of proteins or small DNA binding peptides. Examples include the use of restriction and ligase enzymes as well as small sequence dependent binding peptides for the assembly or disassembly of gold nanoparticles.^80–85^ DNA restriction enzymes have been employed to cleave DNA sites and disassemble gold nanoparticles independently of their DNA melting temperature, and a DNA ligase has been used to “glue” DNA and gold nanoparticles together in a programmable manner. It has also been shown that peptides, which recognise specific DNA sequences, can be bound to gold nanoparticles and drive their self-assembly using DNA.^83,85^

As the field of DNA-nanoparticle self-assembly progresses, new methods to further control the organisation of nanoparticles have become necessary. The scope of this article is to review the progress on the chemical modification of DNA and the use of small molecule intercalators as tools for the manipulation of nanoparticle assemblies. We will first discuss how small-molecule chemical or photochemical DNA modifications (*e.g.* azobenzenes and vinyl-modified bases) as well as DNA intercalators (*e.g.* psoralen) have been used to control the chemical assembly or dissociation of nanoparticles. Then we will focus on rotaxane and catenane DNA structures, which have been employed to create reconfigurable nanoparticle assemblies. Furthermore, we will review DNA backbone modifications (*e.g.* locked nucleic acids, peptide nucleic acids, borane nucleic acids and phosphorothioate DNA) and their role in the stability of nanoparticle self-assemblies.

## 2. Click chemistry DNA modifications

The term “click chemistry” was first introduced in the early 2000s by the Sharpless group to describe a highly specific and selective chemical ligation method between organic molecules.^86^ The main advantages of a click chemistry reaction are that: (i) it is spontaneous, (ii) the product is produced in very high yield, (iii) the reaction is highly selective, stereospecificity is attained, and (iv) it only generates non-reactive byproducts.^86,87^ The most widely employed click reaction remains the copper(i)-catalysed cycloaddition of a generic azide with an alkyne (CuAAC).^88,89^ The alkyne-azide cycloaddition (AAC) reaction was first reported by Huisgen in 1963,^90^ however, its potential remained underexploited until Sharpless and Meldal discovered that the introduction of a Cu(i) catalyst increased the reaction rate by several orders of magnitude.^89,91,92^

**Fig. 1:**
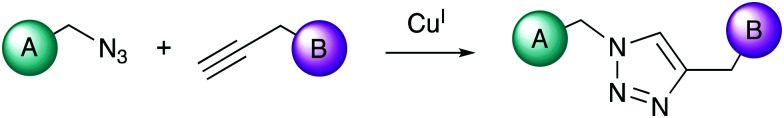
Principle of Cu(i)-catalysed azide–alkyne cycloaddition reaction (CuAAC). Azide functionalised molecule A reacts with alkyne functionalised molecule B in the presence of a copper(i) catalyst forming a 1,4-triazole that covalently links A to B.

The CuAAC reaction forms a 1,4-triazole, a stable 5-membered ring with numerous applications in synthetic chemistry (Fig. 1). Following the introduction of CuAAC, other major groups of click chemistry reactions have been identified that fulfil these criteria, including: (i) various cycloadditions, (ii) nucleophilic ring-openings, (iii) non-aldol carbonyl chemistry, and (iv) carbon multiple bond additions.^93^

The CuAAC reaction is an attractive method for the chemical modification of oligonucleotides. Major advantages are that alkynes and azides can be incorporated into oligonucleotides without disrupting their biological and chemical properties, the alkyne and azide groups are inert under biological conditions, and the final triazole product is non-toxic and extremely stable.

The use of CuAAC with oligonucleotides for the directed assembly of nanoparticles was first demonstrated by Xu and co-workers in 2010,^94^ who developed a quantitative colorimetric assay for the detection of copper(ii) ions. In this study, two batches of 30 nm diameter gold nanoparticles were functionalised with short oligonucleotides terminated with an azide and an alkyne, respectively. The detection system relies on the use of sodium ascorbate to reduce the Cu(ii) ions to Cu(i). Amorphous aggregates were produced by the addition of a third linker strand, complementary to both the alkyne and the azide modified oligonucleotides, yielding a purple suspension (a dark precipitate). In the absence of the appropriate Cu(i) catalyst, the cycloaddition reaction does not take place, and heating up the aggregates above the melting temperature (*T*_m_) of the dsDNA, results in the production of the free oligonucleotide functionalised gold nanoparticles in colloidal suspension (pink colour). However, in the presence of Cu(i), the cycloaddition reaction between the azide and the alkyne functionalised oligonucleotide is rapidly catalysed resulting in the irreversible chemical ligation of the oligonucleotides coated nanoparticles. As a result, heating above the *T*_m_ of the double-stranded DNA (dsDNA) does not lead to the disassembly of the gold nanoparticles, and the solution remains colourless with a purple precipitate. Following appropriate calibration, the shift in the *T*_m_ of the DNA duplex could be directly linked to the concentration of copper ions present.

**Fig. 2:**
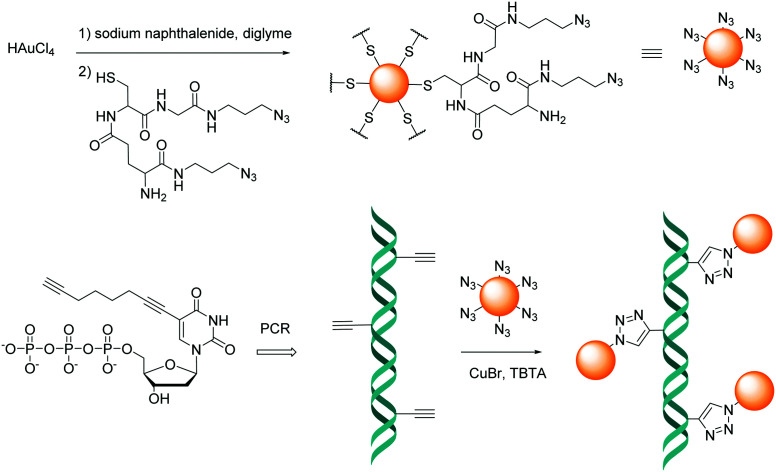
Schematic illustration of the immobilisation of the azide-terminated nanoparticles to alkyne-modified DNA strands *via* a click reaction. TBTA = Tris(benzyltriazolemethyl)amine; PCR = polymerase chain reaction.

The CuAAC method has also been employed to attach nanoparticles to nucleic acid templates. In 2008 Fischler *et al.*^95^ described the synthesis of gold nanoparticles functionalised with a discrete number of glutathione derivatives, modified with an azide moiety. The resulting modified gold nanoparticles were subsequently incubated with a DNA duplex, which was modified with multiple alkyne moieties positioned at regular distances on thymine bases. The formation of triazole linkages involving the dsDNA and the nanoparticles resulted in the formation of one-dimensional chains of particles placed at regular intervals (Fig. 2).

Along with gold nanoparticles, various other inorganic nanoparticles were successfully assembled using click chemistry. For example, Rubner *et al.*^96^ used CuAAC to conjugate oligonucleotides to the surface of upconversion nanoparticles.^96^ The azide and alkyne moieties were placed respectively onto the surface of the upconversion nanoparticles and at the 5′ end of single-stranded DNA (ssDNA), to allow the conjugation between oligonucleotides and upconversion nanoparticles to occur. DNA functionalisation of upconversion nanoparticles enabled the reversible assembly of nanoparticles with complementary DNA strands, enhancing their emission properties.

DNA linkers have also been employed to ligate gold nanoparticles and proteins.^97^ Kendziora *et al.* prepared a novel trifunctional linker to allow multiple orthogonal functionalisation of gold nanoparticles with biomolecules. Thioctic groups on the linker molecule were anchored to the gold nanoparticle surface, while two additional orthogonal functional groups on the same linker molecule were used for the attachment of a heme group and DNA, utilising an amide coupling and the CuAAC reaction, respectively. Using this multifunctional linker, fully functional Au–DNA–Myoglobin hybrids were obtained. Such multifunctional constructs expanded the synthetic toolbox for nanomaterial tailoring, and for design of biosensors and novel catalytic materials.

**Fig. 3:**
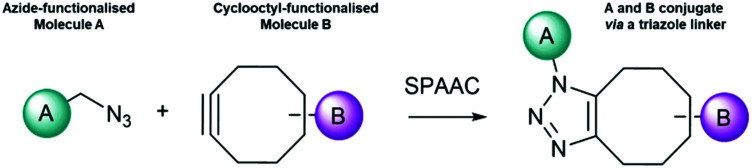
Principle of Strain-Promoted Azide–Alkyne Click Chemistry (SPAAC). Azide functionalised molecule A reacts with cyclooctyne functionalised molecule B yielding a triazole product.

A suitable click chemistry alternative is the [3+2] cycloaddition of azides to cyclooctynes, known as the strain-promoted azide–alkyne cycloaddition (SPAAC). This reaction was first reported in 1961 by Wittig and Krebs,^98^ and the scope has since been greatly expanded and developed. Here, an extremely rapid reaction occurs between a cyclooctyne and an azide (Fig. 3). The driving force of this reaction is the favourable enthalpic release from the ring strain in the cyclooctyne molecule alkyne, (sp-hybridised) becoming an alkene (sp2 hybridised), which leads to the formation of a fused ring system under physiological conditions. Depending on the specific cyclooctyne used, two regioisomers may be formed.

**Fig. 4:**
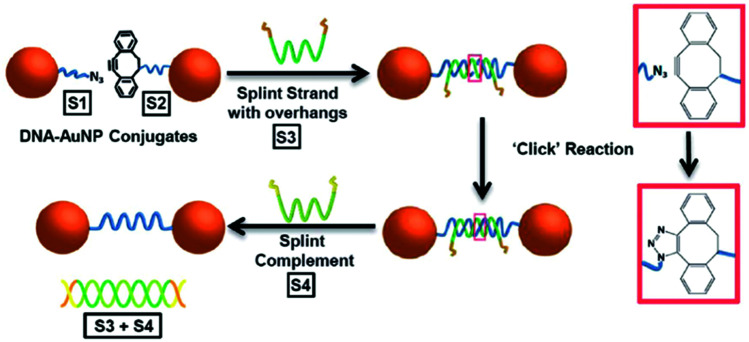
Schematic illustration of gold nanoparticle dimer formation using the DIBO-azide SPAAC reaction. (S1) and (S2) DNA–gold nanoparticle conjugates were brought in proximity by a splint strand (S3). ‘Clicking’ occurred instantly after hybridisation and the addition of DNA strand (S4), resulted in the removal of (S3) through competitive hybridisation. The final nanoparticle dimer system was linked only by a single DNA strand. Reproduced from Heuer-Jungemann *et al.*, *Nanoscale*, 2013, **5**, 7209–7212, with permission from the Royal Society of Chemistry.^100^

The SPAAC reaction was first used to ligate oligonucleotides by Shelbourne *et al.* in 2011.^99^ A ssDNA sequence containing a nucleobase modified with a dibenzocyclooctyne (DIBO) moiety was hybridised to a complementary strand containing a terminal azide facing the DIBO. After hybridisation occurred, the azide and alkyne group were brought into close proximity, allowing for the cycloaddition to occur. The DNA ligation was both rapid and highly specific; even a single base pair mismatch was able to inhibit the reaction rate, suggesting the potential for employing this strategy in multiple simultaneous ligations. In 2013, Heuer-Jungemann *et al.*^100^ extended this strategy to the assembly of oligonucleotide-modified gold nanoparticles. Two batches of gold nanoparticles were functionalised with two different types of ssDNA. Each type of oligonucleotide contained a thiol modification on one end and either an azide (S1) or alkyne (S2) on the other end to facilitate the SPAAC ligation. The addition of a splint strand (S3) complementary to both S1 and S2 templated the formation. Following hybridisation, the DIBO and the azide moieties were placed in proximity and spontaneous covalent ligation occurred. Finally, a fourth oligonucleotide (S4) was incubated with the dimers which removed S3 *via* competitive strand hybridisation (Fig. 4).

**Fig. 5:**
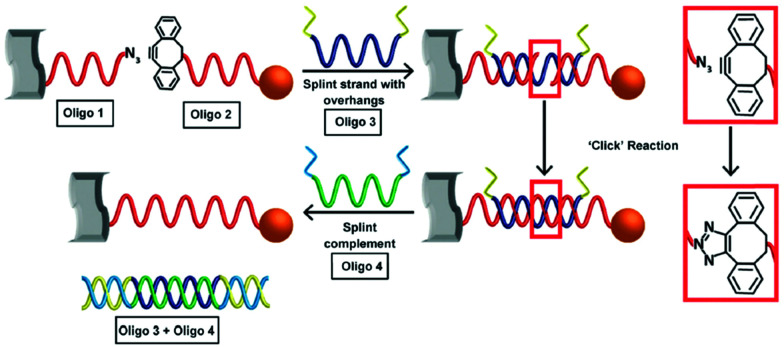
Schematic illustration of the formation of covalently ligated hybrid GO–gold nanoparticle systems. DNA modified graphene oxide and nanoparticles are hybridised with the templating splint strand Oligo 3. After hybridisation, SPAAC occurs rapidly. The addition of a fourth strand removes the templating Oligo 3. Reproduced Heuer-Jungemann *et al.*, *J. Mater. Chem. C*, 2015, **3**, 9379–9384, with permission from the Royal Society of Chemistry.^101^

Using the same strategy, 13 nm gold nanoparticles functionalised with a discrete number of DIBO-modified oligonucleotides were assembled onto the surface of a graphene oxide (GO) nanosheet.^101^ The GO nanosheets were modified with a layer of covalently bound ssDNA functionalised with a terminal azide. After the addition of a templating splint strand, the gold nanoparticles and the GO sheets were assembled *via* three-strand hybridisation. The alkyne and the azide were appropriately positioned for the cycloaddition to occur. After ligation, the splint strand was displaced and removed from the system, leaving the GO–gold nanoparticle hybrids intact (Fig. 5). Furthermore, to demonstrate the formation of more complex hybrid assemblies, a second click ligation step was performed. Small gold nanoparticles (5 nm) were functionalised with a single azide-terminated oligonucleotide and incubated with the GO–gold nanoparticle hybrid. Subsequently, the addition of a splint strand prompted the hybridisation with the larger gold nanoparticles, which allowed the SPAAC reaction to occur, ligating the small gold nanoparticles on the top of the larger gold nanoparticles, demonstrating a new approach for the programming of nanoparticle assemblies on graphene oxide surfaces.

**Fig. 6:**
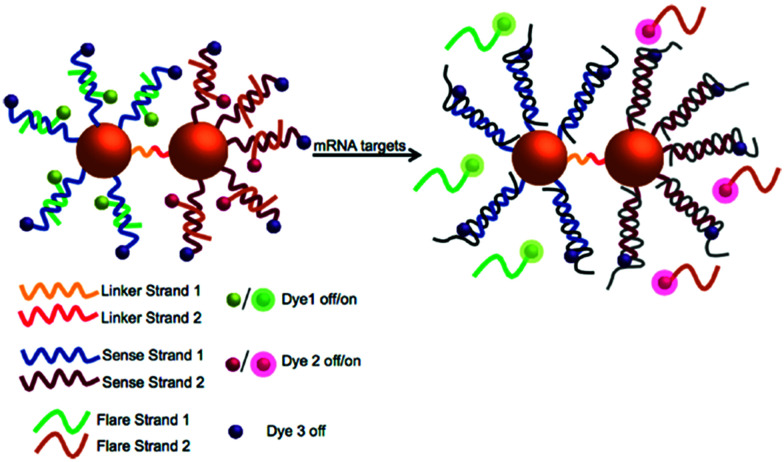
Illustration of the multiplexed nanoparticle dimer ligated by DIBO-azide click chemistry. Two separate batches of gold nanoparticles were functionalised with (linker strand 1) and (linker strand 2) as well as sense strand 1 and sense strand 2 (designed to capture specific mRNA targets). Gold nanoparticle dimers were formed upon hybridisation of linker strands 1 and 2, leading to DNA ligation *via* the SPAAC reaction. Reproduced from Kyriazi *et al. ACS Nano* 2018, **12**(4), 3333–3340, Copyright © 2018 American Chemical Society.^102^

The SPAAC reaction for the ligation of oligonucleotides is exceptionally robust and universal and has been further explored by Kyriazi *et al.*^102^ to fabricate stable nanoparticle dimers for biomedical applications.^102^ Two batches of gold nanoparticles modified with a single oligonucleotide strand (monoconjugates) were prepared. The monoconjugates were chemically modified with either an azide group (linker strand 1) or DIBO (linker strand 2). The gold nanoparticles were functionalised with a shell of sense strands (sense strands 1 and 2), designed to capture specific mRNA targets. Gold nanoparticle dimers were subsequently formed by hybridisation of linker strands 1 and 2, which resulted in spontaneous DNA ligation *via* the SPAAC reaction. These nanoparticle dimers were utilised to sense simultaneously two different mRNA targets within cells and to deliver two different drugs in response to the presence of specific mRNA signatures (Fig. 6).

Employing click chemistry to make stable nanoparticle dimers is of broad interest and it has been recently applied in the fabrication and study of nanoparticle motors on surfaces. Recently, Bazrafshan *et al.*^103^ reported the fastest DNA–gold nanoparticles nanomotors to date, by optimising enzyme and buffer conditions as well as DNA leg span and density. To test whether their nanomotors moved by a rolling motion, they utilised copper-free click chemistry to ligate DNA-coated 50 nm gold nanoparticle to form motor dimers. These dimers demonstrated that nanoparticles motors generated ballistic trajectories, hence they moved predominantly by a rolling mechanism.^103^

The SPAAC oligonucleotide ligation has also been utilised for the fabrication of larger and more complex 3D structures, for example in the assembly of gold nanoparticles on DNA origami. In their 2019 work,^104^ Lin *et al.* used a 3D origami framework to direct the assembly of programmed nanoarchitectures by manipulating directional chemical reactions. An octahedral frame was used to provide up to 6-fold valence through its vertices. The frame was constructed by 12 six-helix bundles. Single-stranded oligonucleotides modified with clickable functional groups (azide or DIBO) were anchored onto the desired vertex of the octahedral frames, providing chemically reactive nanostructures with directionally defined valence. By choosing structures with desired valence for click reaction, different frames with controlled geometries could be generated, including dimer, trimer, cross-shaped, and chain nanostructures. Additionally, frames functionalised with DIBO at specific vertices were combined with gold nanoparticles involving a dense shell of azide-modified oligonucleotides. Upon mixing, the SPAAC reaction occurred between the frames and the gold nanoparticles, allowing the specific arrangement of the nanoparticles in pre-programmed positions on the octahedral frame.

Overall, click chemistry is an attractive tool for the programmable manipulation of DNA-driven nanoparticle self-assembly. It is fast and efficient, leading to a high yield of a stable triazole linkage, and it occurs at room temperature in aqueous solvents. Furthermore, the copper-free click chemistry strategy has a number of additional advantages. The dibenzocyclooctyne and azide moieties display high reaction specificity under optimised conditions, even in the presence of common chemical groups and complex environments. Additionally, no catalyst is required, which, combined with the mild conditions required for the conjugation, renders it suitable for building macromolecular assemblies. However, when considering click chemistry strategies for the chemical ligation of nanoparticles assemblies, a possible limitation is posed by the fact that the reaction between azide and alkyne occurs rapidly and irreversibly, leaving little room for post-synthetic rearrangement of nanoparticles.

## 3. Cyclobutane pyrimidine DNA modifications

Light is an attractive stimulus to manipulate nanoparticle self-assembly, as it is highly specific and does not introduce contaminants. In addition, the characteristics of light can be easily tuned by controlling the intensity and wavelength of the illumination. DNA self-assembled nanostructures can be rendered responsive to light in two different ways: either by intercalating photoactive molecules within a DNA duplex, or by chemically modifying nucleic acids with light-responsive molecules. In both cases, irradiation with light can trigger photochemical reactions within DNA nanostructures that can result in chemical ligation or DNA dehybridisation.

The most commonly used photo-ligation strategy relies on the [2+2] photocycloaddition between two alkenes. Following the promotion of one of the alkenes to an excited state, the rearrangement of the electrons leads to the formation of two new bonds. This reaction has been known since the late 1800s^105^ and has been widely exploited for a variety of applications. The thorough description of this type of reactions and the general applicability is beyond the scope of this work and dedicated reviews are available.^106^

In this section we will focus our attention on DNA photo-chemical modifications, which have been employed for the manipulation of DNA-nanoparticle assemblies. We will discuss modifications that can be introduced as intercalating agents within DNA duplexes, such as psoralen-based intercalators, and describe the use of photoactive nucleobases within DNA sequences including vinyl modifications. Azobenzene compounds, a widely used class of photoactive molecules, will be discussed in a separate section due to the larger volume of relevant studies.

### 3.1. Vinyl DNA modifications

Oligonucleotides with electron-withdrawing vinyl modifications photo-react in the presence of an adjacent pyrimidine nucleobase (cytosine, thymine, or uracil) and form a cyclobutane ring between complementary oligonucleotides *via* a [2+2] photocycloaddition. These modifications have been extensively explored and optimised for use in complex DNA systems, such as logic circuits, by the Fujimoto group.^107–110^ The inclusion of electron-withdrawing groups (for example, carboxyl and cyano- moieties) further enhances reactivity;^111,112^ enabling light manipulation of DNA structures, even for highly complex origami assemblies.^113^

**Fig. 7:**
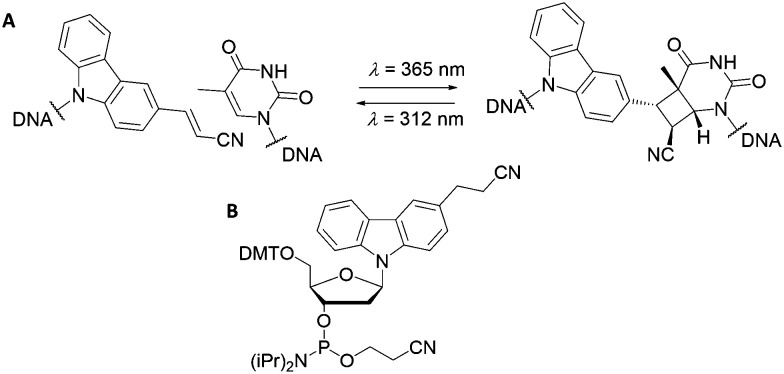
(A) Reversible photocycloaddition between a cyanovinyl carbazole and a thymine base. (B) Molecular structure of the commercially available cyanovinyl carbazole phosphoroamidite.

The cyanovinyl carbazole nucleoside is a commonly used photo-crosslinking modification in the field of nucleic acid chemistry. The Fujimoto group first introduced a cyanovinyl carbazole nucleoside into a nucleic acid sequence using the phosphoramidite shown in Fig. 7, which is now commercially available. The subsequent light-sensitivity was tested when the modification was positioned opposite thymine, guanine, adenine or cytosine (T, G, A or C) in the complementary strand and irradiated with UV light at a wavelength of 365 nm.^114,115^ The fastest and highest yielding reaction occurred when a thymine nucleobase was positioned diagonally, opposite in the complementary strand (Fig. 7). This photo-crosslinking reaction is both clean (occurring without the addition of external reagents) and fast (with an irradiation time of only 1 s). The reaction can be reversed by UV light irradiation at 312 nm wavelength, opening the cyclobutane ring and to yield the cyano-ethylene group and thymine base in the unbound configuration.

**Fig. 8:**
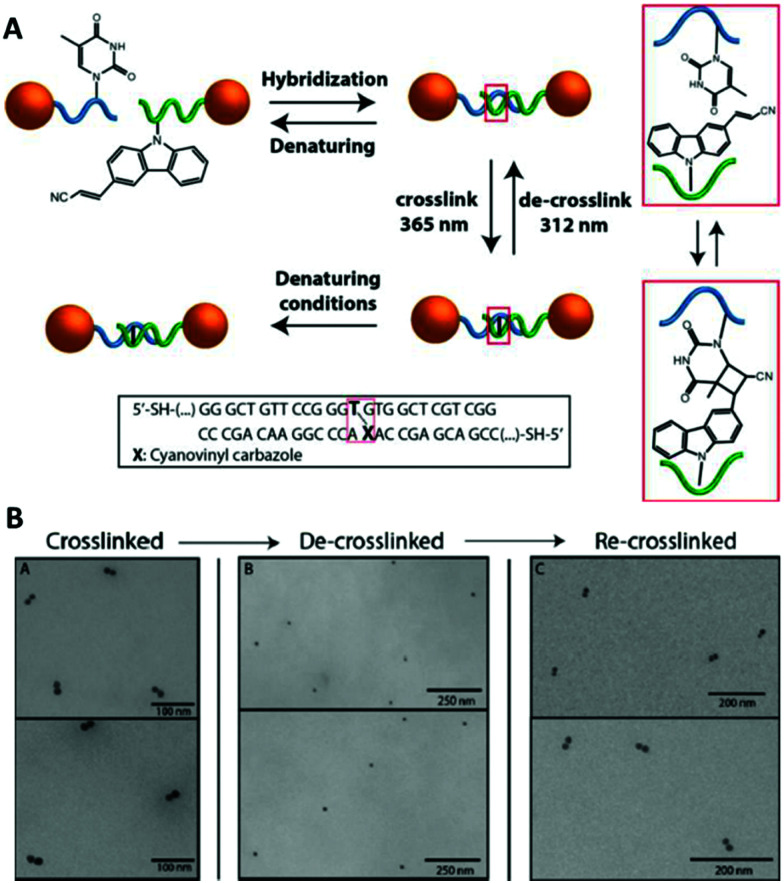
(A) Schematic illustration of the reversible photoligation between DNA-nanoparticle dimers. The cyanovinyl carbazole group is incorporated in one of the DNA sequences, diagonally facing a thymine base located in the opposite strand. Upon irradiation with 365 nm light, a cyclobutane bridge between the strands is formed (red box). After crosslinking, the dimers remain intact even under DNA denaturing conditions. The ligated DNA strands can be reversibly de-crosslinked upon light irradiation at a wavelength of 312 nm. (B) TEM micrographs of gold nanoparticles dimers after crosslinking/decrosslinking light and heat cycles. Reprinted with permission from Harimech *et al.*, *J. Am. Chem. Soc.* 2015, **137**(29), 9242–9245, copyright (2015) American Chemical Society.^116^

The cyanovinyl carbazole group was first used in 2015 as a DNA modification to manipulate the assembly of DNA-functionalised nanoparticles.^116^ In this paper, Harimech *et al.* used 15 nm gold nanoparticles functionalised with a discrete number of oligonucleotides to build dimers, trimers and tetramers assemblies of gold nanoparticles *via* DNA hybridisation. One DNA strand was chemically modified with a cyanovinyl carbazole nucleoside, and the complementary DNA strand was designed in such a way that after hybridisation occurred, a thymine group was positioned diagonally, opposite the cyanovinyl carbazole group. By irradiating with 365 nm wavelength light, the DNA gold nanoparticle oligomers were ligated (Fig. 8), enhancing their stability under DNA denaturing conditions. In addition, the authors demonstrated the reversibility of the crosslinking reaction by irradiating the DNA duplex with 312 nm wavelength light. This approach allowed the efficient and reversible ligation of gold nanoparticles without the use of additional reagents, and without causing any DNA damage under the mild conditions of UV irradiation chosen.

**Fig. 9:**
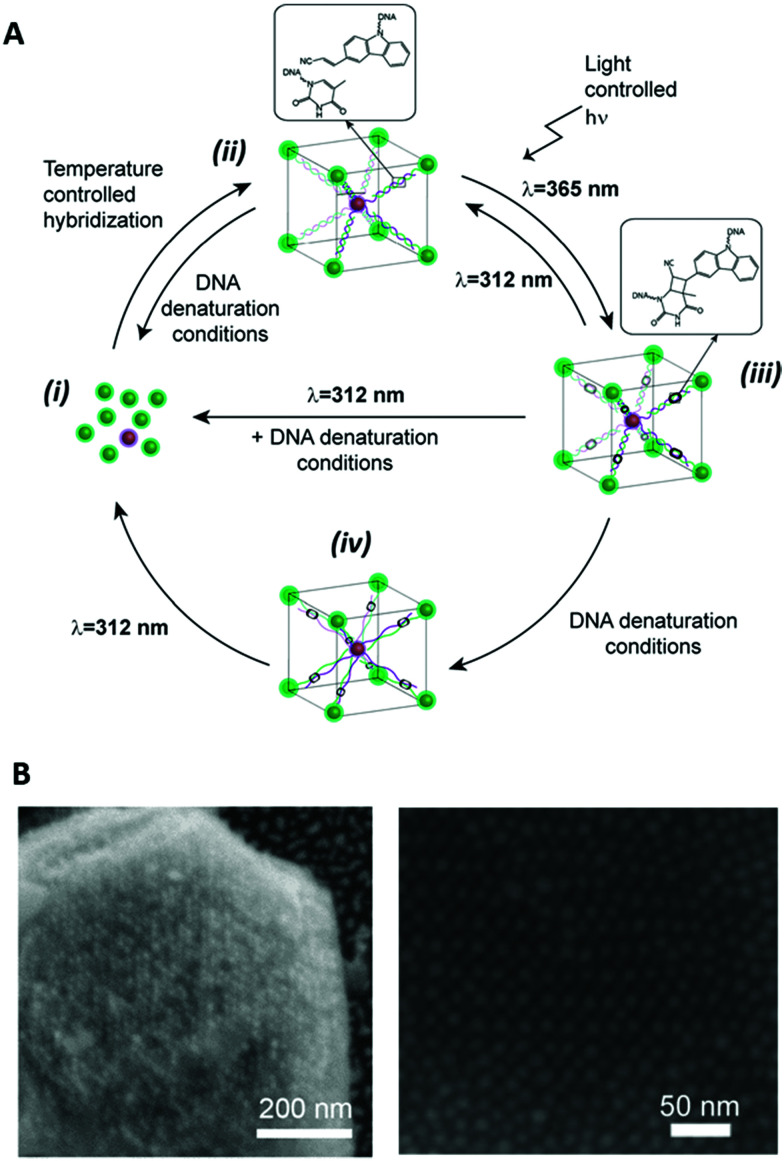
(A) Schematic illustration of the reversible photoligation of 3D superlattices. nanoparticles conjugated cyanovinyl carbazole modified oligonucleotides, hybridised under thermal controlled conditions to form nanoparticle superlattices. The oligonucleotides photoreacted upon light irradiation at 365 nm to form interstrand chemical bonds between the cyanovinyl carbazole and an adjacent thymine in the complementary strand. This photochemical process was reversed upon irradiation with light at 312 nm. (B) SEM micrographs of body centred cubic gold nanoparticles superlattices. Reproduced with permission from De Fazio *et al.*, *ACS Nano* 2019, **13**(5), 5771–5777, copyright (2019) American Chemical Society.^117^

Recently, De Fazio *et al.* demonstrated that light can be employed as an external stimulus to master the assembly of extended 3D nanoparticle superlattices.^117^ In this work, carbazole-modified oligonucleotides were employed to fabricate micron-sized crystal structures by thermal control of the DNA hybridisation process.^58^ The carbazole-modified nucleobases remained unreactive throughout the crystallisation process, without hindering the formation of ordered arrays of nanoparticles. After crystal formation, the superlattices were irradiated with 365 nm light to activate the carbazole and trigger the covalent ligation. To prove the successful formation of crosslinked 3D crystals, the superlattices were transferred to DNA denaturing conditions, which did not degrade the nanoparticle superlattices. To demonstrate the reversibility of this approach, the gold nanoparticle superlattices were disassembled by irradiation at 312 nm and subsequent transfer to DNA-denaturing conditions caused the breaking of the covalent bond and the disassociation of the complementary oligonucleotides (Fig. 9).

**Fig. 10:**
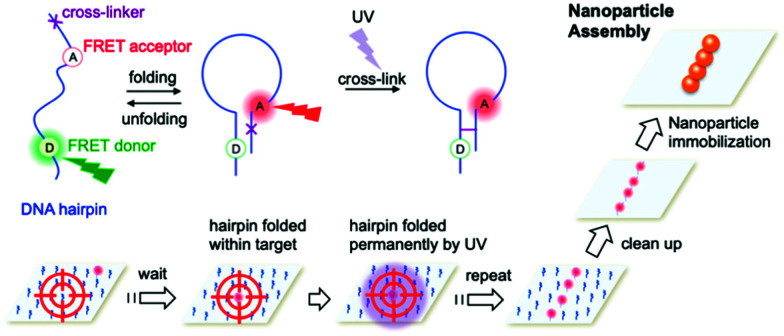
Nanoparticle assembly using super-resolution optical lithography with DNA. Hairpins deposited on a microscope slide randomly fold and unfold, resulting in stochastic FRET acceptor blinking within the FRET illumination area. When a hairpin folds within a target spot, it is irradiated with UV light, resulting in the hairpin crosslinking in the folded structure. After completing the crosslinking in a designated pattern, the slide is incubated with nanoparticles coated with a short strand complementary to the hairpin sticky ends and immobilised to form the target assembly. Reproduced with permission from Kim *et al.*, *Nano Lett.* 2019, **19**(9), 6035–6042, copyright © (2019) American Chemical Society.^118^

Cyanovinyl carbazole has also been employed in two-dimensional (2D) lithography fabrication techniques. Kim *et al.*^118^ showed the efficacy of this modification by developing a super-resolution microscopy method to assemble nanoparticles employing a three-strand hairpin construct modified with a carbazole crosslinker. Selective UV crosslinking occurred only when the hairpins were found in their folded state, allowing the hairpin strand to remain in the folded state, even upon ‘warm’ washing (above the *T*_m_ of the DNA hybridisation). After the selective crosslinking process and subsequent cleaning, nanoparticles coated with the effector DNA strand complementary to the pin strand were immobilised only on the crosslinked hairpins. The hairpins were also functionalised with dyes signalling the hybridisation into the folded state. The stochastic fluorescence blinking due to the spontaneous folding and unfolding motions of DNA hairpins enabled the precise localisation of a folded hairpin and solidification only when within a predesigned target area (Fig. 10).

In addition to the development of the cyanovinyl carbazole, Fujimoto's group evaluated the commercially available 5-carboxyvinyl-2′-deoxyuridine as a photo-crosslinking modification.^108^ They demonstrated that 5-carboxyvinyl-2′-deoxyuridine could be used to photo-ligate two ends of an oligonucleotide to form a cyclic oligonucleotide. By irradiating at 366 nm, these artificial nucleotides produced photo-ligated catenated sequences with high efficiency without any side reactions. Irradiation at 312 nm resulted in the photo-ligated nucleic acids reverting to their original form.

The 5-carboxyvinyl-2′-deoxyuridine photo-crosslinking modification has been exploited in a gold nanoparticle-based surface enhanced Raman scattering (SERS) based DNA sensor.^110^ In this probe two oligonucleotide functionalised gold nanoparticles were used, one with terminal 5-carboxyvinyl-2′-deoxyuridine nucleoside and the other with a terminal uracil. The two gold nanoparticles hybridised against a target DNA and photo-irradiation was used to covalently crosslink the DNA between the gold nanoparticles leading to the formation of stable nanoparticle assemblies. Once the nanoparticle assemblies were formed, the space between adjacent gold nanoparticles acted as a stable “SERS hot spot”. Therefore, in the presence of a target DNA, a SERS signal from Raman-active molecules (sodium cacodylate) was easily detected. In contrast, a SERS signal was not detected if the DNA target was not present. A major advantage of this sensor was its simplicity since it did not involve enzymatic reactions, fluorescent dyes, precise temperature control, or complicated operating procedures.

Another electron-withdrawing vinyl variation is the *p*-carbamoylvinyl phenol nucleoside (also referred to as cinnamate).^119^ An oligonucleotide containing such a moiety can be crosslinked with adjacent adenine residue in a [2+2] fashion by exposure to 366 nm light; however, if two cinnamate residues are placed in complementary strands facing each other, they can also selectively crosslink upon illumination in the UV light range of 360–390 nm. This method was employed by Feng *et al.*^120^ to develop a photolithography method for the patterning of DNA-functionalised polystyrene particles onto surfaces down to 1 μm resolution.

The work described above showed that electron-withdrawing vinyl modifications are extremely versatile and widely applicable in various contexts, from small oligomers and bi-dimensional platforms to the formation of extended crystals. One of the main advantages of using vinyl modifications is the extra robustness provided to the resulting DNA-nanoparticle structures, without the introduction of external reagents or contaminants in the reaction vessel. Furthermore, the vinyl modification can be positioned within the oligonucleotide sequence with high specificity, allowing for complete control over the site of crosslinking. In addition, the cyanovinyl carbazole and the carboxyvinyl-2′-deoxyuridine modifications provide chemical reversibility; the chemical bonds can be broken to yield the pristine oligonucleotides. This is an advantage for the fabrication of dynamically controlled structures, as it provides greater stability in comparison with unmodified DNA strands without compromising the flexibility provided by DNA. However, it should be noted that the light-active nucleobases must be inserted within the oligonucleotide sequence during the synthesis, which requires a careful pre-design. As a result, the addition of modified nucleobases can be expensive and sometimes leads to low yields. To maximise the photo-crosslinking reaction, one must ensure that the whole sample is exposed to light, thus the irradiation conditions (total power, intensity per area and distance from the light source) need to be optimised.

### 3.2. Psoralen derivatives

**Fig. 11:**
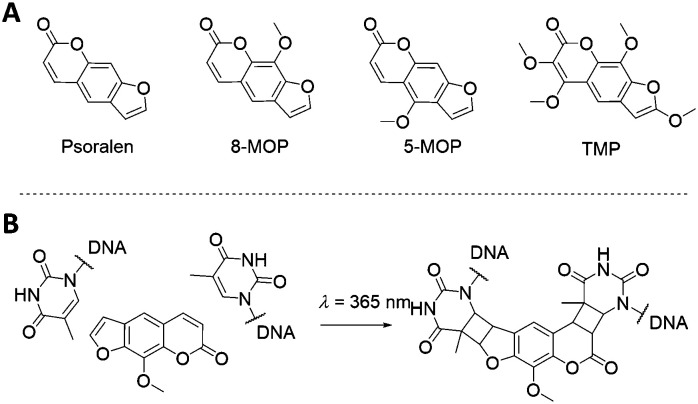
(A) Psoralen-related molecules. Psoralen, 8-methoxypsoralen (8-MOP), 5-methoxypsoralen (5-MOP) and 4,5′,8-trimethylpsoralen (TMP) are of particular interest for DNA-guided nanoparticles self-assembly. (B) Illustration of the intercalation and crosslink of an 8-MOP molecule between two thymine residues.

The psoralen aromatic system represents another class of photoactive molecules (Fig. 11). Psoralen is a bi-functional molecule that can be used as a photoactive probe of nucleic acid structure and function. Psoralen intercalates within dsDNA and upon exposure to UV irradiation (320–400 nm wavelength) can form pyrimidine-psoralen mono- and di-adducts with adjacently stacked pyrimidine bases. The key step of the mechanism is the [2+2] cycloaddition photoreaction between one of the two reactive double bonds of psoralen and the reactive C5=C6 double bond of thymine, leading to a cyclobutane structure, linking both molecules. The photochemical properties of psoralen have been known for over 40 years^121^ and can be exploited to generate DNA probes containing psoralen mono-adducts at specific sites that prevent the division of injured cells. This mechanism is at the core of the so-called PUVA (Psoralen + UV-A) therapy, and it has been explored for a wide range of applications, from skin cancer to blindness treatments.^122–126^ In recent years, psoralen has been utilised to stabilise extended DNA origami structures.^127,128^

One of the earliest examples that incorporated psoralen-derivatives into DNA templates to direct the assembly of nanoparticles was reported by Patolsky *et al.*^129^ In their 2002 study, they demonstrated the generation of gold nanoparticle wires onto DNA templates. The DNA–gold nanoparticle wires were fabricated by the incorporation of intercalator-functionalised gold nanoparticles into dsDNA, followed by the photochemical cross-linking of the intercalator to the DNA matrix. The intercalating agent, an amino psoralen moiety, was directly attached on the surface of 1.4 nm gold nanoparticles functionalised with a single *N*-hydroxysuccinimide ester group, forming an amide bond between the psoralen and the gold nanoparticle. The modified gold nanoparticles were then mixed with a double-stranded poly A/poly T duplex, and the resulting assembly was irradiated with a *λ* > 360 nm UV light. These conditions induced the [2+2] cycloaddition between the psoralen groups and the thymine residues of the DNA template, leading to the covalent attachment of the intercalator-functionalised gold nanoparticles to the DNA.

A DNA template was also used in the work carried out by Li *et al.*^130^ They produced a chemically crosslinked branched DNA nanostructure with the aim of creating a recyclable biosensing platform for highly specific and ultra-sensitive DNA detection. This detection system consisted of a crosslinked DNA probe for the recognition of a target DNA, coupled with DNA-functionalised iron oxide (Fe_3_O_4_) nanoparticles for signal amplification. A high density of thymine bases was included in the double-stranded segment of the branched DNA to enable photo-crosslinking with psoralen molecules. Owing to the presence of multiple covalent bonds, this biosensing platform endured denaturation and high-temperature conditions, allowing regeneration of the interface. The device could be recycled without loss of detection sensitivity.^130^

**Fig. 12:**
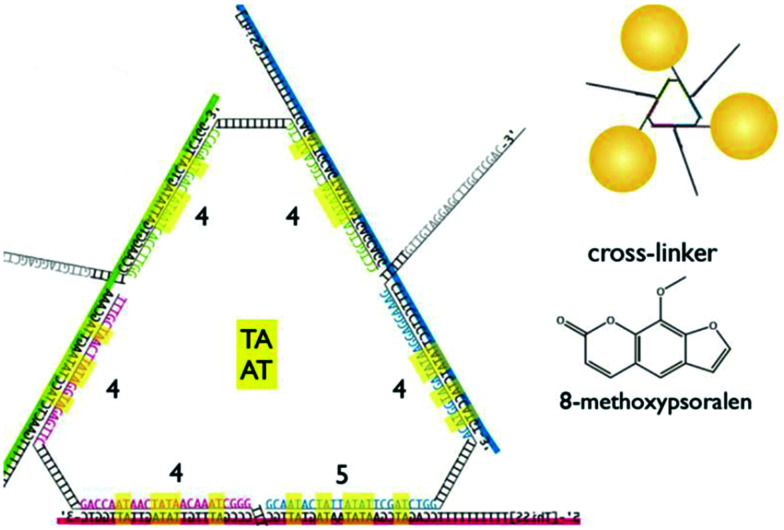
Triangular assembly of gold nanoparticles, obtained by intercalation of 8-methoxypsoralen. The DNA template was designed to have twenty-five 5′-TA-3′ sites as suitable positions for intercalation and subsequent crosslinking, resulting in stable formation of triangular assemblies. Reprinted from Ohshiro *et al.*, *Chem. Commun.*, 2010, **46**, 6132–6134 with permission from The Royal Society of Chemistry.^131^

An alternative approach is to directly attach synthetic oligonucleotides on the surface of nanoparticles and assemble these *via* hybridisation with complementary strands prior to addition and photo-crosslinking using psoralen. Through this strategy, both discrete and extended nanoparticles arrays have been successfully covalently crosslinked. Ohshiro *et al.*^131^ used this method to direct the cyclic assembly of gold nanoparticle trimers and tetramers using a DNA template. In their work, they functionalised the surface of 20 nm gold nanoparticles with a discrete number of thiolated-ssDNA strands and, following the addition of a DNA template, they generated cyclic oligomers comprising of three or four gold nanoparticles. These structures were then treated with 8-methoxypsoralen (8-MOP) which intercalated into the duplexes, and then irradiated with 365 nm light to activate the reaction at the TATA region of the linker DNA (Fig. 12). This approach provided control of both the number and the geometry of gold nanoparticles, as well as ensuring extra stability and accurate positioning of the 8-MOP at the TATA coding regions.

Using a similar strategy, Xie *et al.*^132^ developed a colorimetric method for the detection of psoralens (anticancer intercalating agents) based on the combination of self-assembly of oligonucleotide-modified gold nanoparticles and inter-strand crosslinking. After the fabrication of DNA–gold nanoparticles dimers, they diffused different psoralen intercalating agents into the dimer suspension, followed by 365 nm irradiation. To prove the effectiveness of the covalent ligation, the crosslinked dimers were heated above the dsDNA *T*_m_. The dimers remained intact, proving the successful inter-strand crosslinking of TMP and 8-MOP. Using the same method, Lee *et al.*^133^ reported the fabrication of an extended array of DNA-functionalised gold nanoparticles, covalently bound by 8-MOP intercalators. UV crosslinking was used to stabilise a 3D gold nanoparticle crystal, which presented the additional challenge of achieving a sufficient penetration depth of the light. The first step consisted of the controlled assembly of DNA-coated gold nanoparticles into 3D crystalline structures, followed by the permeation of intercalating molecules. The irradiation with UV light irreversibly crosslinked the DNA duplexes maintaining the lattice network, thus stabilising it against a variety of DNA-destabilising conditions. In this same report, the capability of bis-chloroethyl nitrosourea to covalently bind cytosine and guanine residues was also explored.

Although the vinyl-modified nucleobases are amongst the most commonly used chemical modifications for photo-crosslinking oligonucleotides, the use of psoralen derivatives presents a significant advantage in terms of versatility, as it can be introduced in any oligonucleotide system, both *a priori* in the design and synthesis of the oligonucleotides, and *a posteriori* as an intercalator during the hybridisation phase. This feature renders psoralen modifications extremely versatile and easily applicable. However, one must consider that the positioning of the intercalator may not be as precise as in the case of a modified nucleobase and the exact number of intercalated molecules is often unknown. Additionally, the DNA crosslinking utilising psoralen is typically an irreversible process.^134^

### 3.3. Azobenzene derivatives

**Fig. 13:**
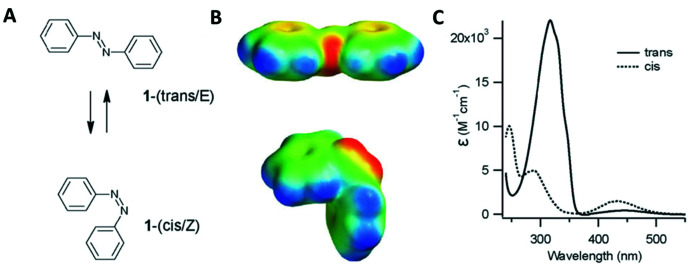
(A) *E* (*trans*) and *Z* (*cis*) structural configurations of the azobenzene molecules. (B) Corresponding electrostatic potentials. Red indicates negative potential and blue indicates a positive potential. (C) Electronic absorption spectra for the *trans* (solid line) and *cis* (dotted line) configurations. Adapted from Beharry *et al.*, *Chem. Soc. Rev.*, 2011, **40**, 4422–4437, with permission from The Royal Society of Chemistry.^137^

Azobenzene compounds were first discovered in the mid-1800s. Fig. 13 shows the chemical structure and absorption spectra of azobenzene molecules. They contain two phenyl rings, which are separated by a –N=N– (azo) bond and represent the parent molecule for a wide range of aromatic azo compounds, which are primarily used in the dye industry.^135–137^ One of the most interesting characteristics of azo compounds is the quickly induced and reversible *trans*–*cis* photoisomerisation of the azo bond as well as the structural changes occurring when they are integrated within other materials.^138,139^ This interconversion is induced by light (at specific wavelengths), which renders azo compounds excellent candidates for photo-switching activity effectively controlling the optical, chemical, mechanical and electronic properties of materials.^138^

Azobenzene compounds are the most commonly used moieties for the production of photo-responsive oligonucleotides. This is attributed to: (i) their ease of synthesis; (ii) high quantum yields; and (iii) efficient/quick photo-switching activity.^140^ Asanuma and colleagues pioneered the synthesis and characterisation of oligonucleotides involving azobenzene modifications. In 1999, they demonstrated that photoisomerisation using UV or visible light could be used to control the hybridisation (or melting) of a DNA duplex modified with azobenzene.^141^ The introduction of azo moieties in DNA fragments has enabled the construction of dynamic systems (for example, multidirectional bi-dimensional origami structures).^142^ The development of azobenzene compounds for the construction of photo-responsive oligonucleotides has been extensively reviewed by Lubbe and colleagues.^140^

Azobenzenes have also been extensively used for the functionalisation of various types of nanoparticles and their subsequent controllable assembly/disassembly. For example, when exposed to visible light (*λ* = 430–460 nm), *cis*-to-*trans* isomerisation of the azobenzene occurs, leading to the loss of the nanoparticles’ colloidal stability and their subsequent assembly. However, when exposed to UV light irradiation (*λ* = 300–370 nm), a *trans*-to-*cis* isomerisation of the azobenzene is induced and as a result, the re-dispersion of nanoparticles occurs.^143^ Assembly and disassembly of nanoparticles can occur multiple times owning to the chemical robustness of azobenzene compounds.^35^

In an early study conducted by Han and co-workers, reconfigurable 3D DNA nanostructures were constructed using azobenzene modified DNA.^144^ Initially, DNA tetrahedral structures were assembled with a DNA hairpin structure integrated within the DNA tetrahedron. The hairpin could be opened or closed by hybridisation with an azobenzene modified DNA strand. The azobenzene modified strand can only hybridise to and open the hairpin structure when the azobenzenes are in the *trans* configuration. When irradiated with UV light (*λ* = 350 nm), the azobenzene molecules assume the *cis* configuration and the modified strands dehybridised from the tetrahedral structure allowing the DNA hairpin to reform and shortening one edge of the tetrahedron. Exposure to visible light (*λ* = 450 nm), resulted in a change in the configuration of the azobenzene to *trans* and the azobenzene-modified DNA strand hybridises to the hairpin region, returning the tetrahedron to its original shape. In order to observe these structural changes, 3.5 nm gold nanoparticles were assembled onto the DNA tetrahedral nanostructures. Transmission electron microscopy (TEM) revealed a change in the distance of the gold nanoparticles when the structures were irradiated with UV light. Prior to UV irradiation, the two isosceles edges were approximately 8 nm long, while the bottom edge was 11 nm long. Following UV light exposure, the DNA tetrahedral structures contracted, and the bottom edges of the triangles decreased to 4 nm.

**Fig. 14:**
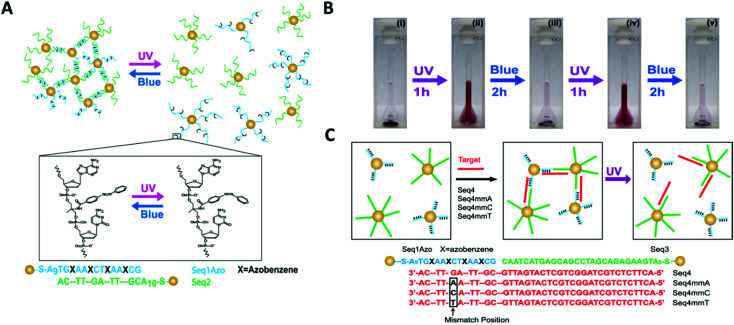
(A) Schematic illustration of gold nanoparticles functionalised with azobenzene-modified DNA. Hybridisation of nanoparticles bearing complementary sequences is controlled by illumination with UV and blue light *via trans–cis* photoisomerisation of azobenzene. In (B) digital photographs of the assembly and disassembly of the light-responsive gold nanoparticle conjugates following irradiation with UV and blue light. (C) shows that photoswitchable assemblies could be used to discriminate single-base mismatches. Reproduced with permission from *Nano Lett.* 2012, **12**(5), 2530–2536, copyright © (2012) American Chemical Society.^145^

Yan and colleagues reported a novel class of light-responsive metallic nanoparticle assemblies, comprised of 15 nm gold nanoparticles functionalised with azobenzene modified oligonucleotides.^145^ Whilst photo-regulation of gold nanoparticles assemblies with various types of azobenzene containing ligands has been previously reported,^146–148^ this approach employed the specificity of azobenzene modified oligonucleotides to accurately manipulate the gold nanoparticles self-organisation. Gold nanoparticles were functionalised with DNA strands containing four evenly spaced azobenzene modifications, which formed assemblies when mixed with gold nanoparticles functionalised with the complementary DNA sequence. The resulting gold nanoparticle assemblies were able to assemble and disassemble using light, upon the azobenzene configurational change from *trans* to *cis* (Fig. 14A–C).

The study also demonstrated that light could be used instead of temperature to distinguish between complementary sequences and sequences that contained a single base mismatch. Therefore, the light-induced melting properties of these gold nanoparticles assemblies could potentially be used to verify the presence of specific targets or to detect the presence of single point mutations that are linked to disease. Further possible applications could include the development of new sensing platforms by speeding up the analysis process, minimising the complexity of DNA hybridisation assays that use microfluidic devices, or by introducing a pump/probe-based sensor system.

**Fig. 15:**
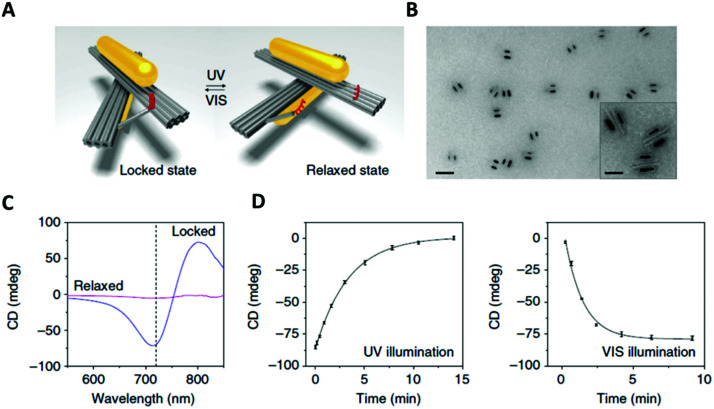
(A) 3D reconfigurable plasmonic DNA origami nano-construct regulated by UV and visible light irradiation. Red region indicates the azobenzene-modified oligonucleotides. (B) TEM images of the 3D plasmonic DNA origami nano-constructs in the locked right-handed configuration. Scale bars at 50 nm in the inset and 200 nm in the large image. (C) CD spectra following UV and blue light irradiation. (D) Kinetic characterisation of the 3D plasmonic DNA origami nano-constructs reverting from the locked right-handed state to the relaxed state and *vice versa* upon UV and blue light irradiation. Reprinted with permission from *Nat. Commun.*, 2016, **7**, 10591. Permission granted *via* Creative Commons CC. Copyright © 2016 Springer Nature.^149^

An original approach to manufacturing a light-responsive, reconfigurable DNA–gold nanoparticles plasmonic nanostructure was reported in 2016 by Kuzyk and colleagues.^149^ Optically controlled assemblies made of DNA origami gold nanorods were constructed. More specifically, the gold nanorods (38 nm × 10 nm, in size) were assembled on a reconfigurable DNA origami template made of two 14-helix bundles assembled in a cylindrical shape (80 nm × 16 nm × 8 nm). An azobenzene-modified DNA switch was introduced to enable light stimulation. This photo-responsive segment consisted of two DNA branches, protruding from the two origami bundles (Fig. 15A, red strands). One branch had a ssDNA with 3 azobenzene-modified oligonucleotides, while the other branch contained 4 azobenzene molecules. The two branches were pseudo-complementary and, through appropriate light stimulation, the azobenzene moieties could be isomerised between the *cis* and *trans* configuration. Following this transition, the two hanging ssDNA could be selectively hybridised and de-hybridised (Fig. 15). In this system, light was also employed to induce a plasmonic response in the gold nanorods assembled on top of the two origami bundles.

*In situ* monitoring of the dynamic conformational changes induced by light, was carried out by circular dichroism (CD) spectroscopy. The CD responses were recorded during UV (*λ* = 365 nm) and visible (*λ* = 450 nm) light irradiation. After visible light irradiation, the 3D chiral plasmonic nanostructure accessed a locked state and the CD response produced was characteristic of a right-handed state (Fig. 15C). Following UV light irradiation, the 3D chiral plasmonic nanostructure reverted back into a relaxed state and the resulting CD spectrum significantly decreased and the right-handed form signal was ‘erased’. In addition, their proposed system had the ability to intensify the structural changes of azobenzene due to the use of gold nanorods and as a result translate those light responses into reversible plasmonic chiroptical ones. This 3D chiral plasmonic nanostructure may initiate a new platform for sensing applications while by collecting light energy, these structures could potentially produce collective responses, which can then be translated into controlled molecular motions up to a macroscopic level.

**Fig. 16:**
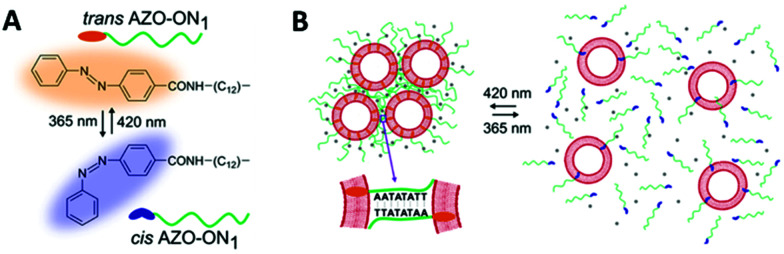
Schematic illustration of reversible assembly/disassembly of unilamellar lipid vesicles, induced by light. (A) Chemical structure of AZO anchor in its *trans* (orange) and *cis* (blue) isomers. (B) Unilamellar lipid vesicles are shown as red circles and the azobenzene functionalised oligonucleotides are represented by the green lines. On the left, at 420 nm light irradiation, assembly occurs and the azobenzene is found at the *trans* configuration (orange ending green lines). Similarly, on the right, at 365 nm light irradiation, disassembly occurs and the azobenzene is found at the *cis* configuration (blue ending green lines). Reproduced with permission from *Nano Lett.*, 2016, **16**(7), 4462–4466, Copyright © 2016 American Chemical Society.^150^

Hernández-Ainsa and co-workers demonstrated the reversible assembly of lipid unilamellar vesicles (LUVs) using azobenzene-modified DNA molecules.^150^ In detail, a self-complementary oligonucleotide was modified with a hydrophobic anchor made of an aliphatic chain with an azobenzene at the end. The modified oligonucleotide was added to a solution of large LUVs, 200 nm in diameter, inducing LUVs assembly (Fig. 16). Then, the effect of the azobenzene isomerisation in the LUV assembly was investigated. The *cis*–*trans* isomerisation was accompanied by a modification in the polarity of the azobenzene moieties, which influenced the hydrophobicity meaning that the strength of the oligonucleotide interaction with the LUVs could be controlled, altering the assembly behaviour of the LUVs. When irradiating with a wavelength of 365 nm the disassembly process was induced. Interestingly, reassembly could be produced by irradiating at 420 nm. This assembly/disassembly process was confirmed to be stable for up to 4 cycles. Additionally, the authors showed that the reversible assembly could also be triggered by ionic (Mg^2+^) and temperature stimuli. Hence, this multi-responsive assembly approach represents a very promising strategy for the development of reversible assemblies, whose properties can be simultaneously controlled by multiple stimuli.

**Fig. 17:**
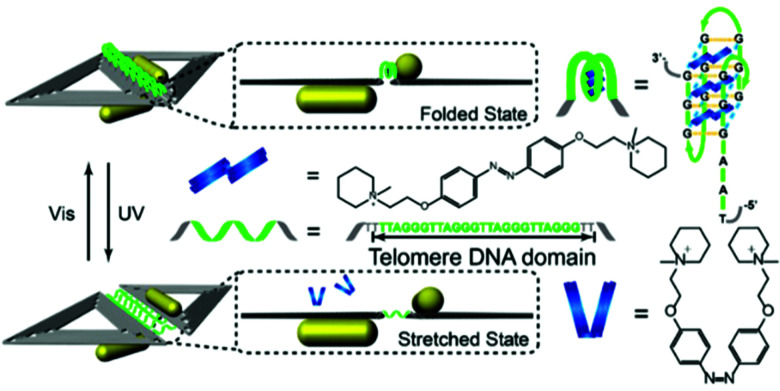
Schematic illustration of the photo-response mechanism. The controller strands (green) contain telomere DNA sequences and form a G-quadruplex with the azobenzene moiety (blue). Following illumination with UV light, the *cis* form of Azo dissociates from the telomere DNA controller strands, and the folded G-quadruplex becomes stretched, resulting in an increased distance between the nanorods. Upon irradiation with visible light, the azobenzene converts into its *trans* isomer, inducing the controller DNA to refold into the G-quadruplex, leading to smaller inter-nanorod separation. Reprinted with permission from *Nano Lett.*, 2017, **17**(11), 7125–7130, Copyright© 2017 American Chemical Society.^151^

Jiang and co-workers reported the use of light susceptible DNA origami structures for the assembly of plasmonic particles.^151^ They demonstrated the construction of stimuli-responsive chiral plasmonic nanostructures by assembling gold nanorods (40 nm × 12 nm in size) into L-shaped configurations utilising rhombus-shaped DNA origami templates (Fig. 17).

In total, nine telomeric ssDNA sequences were used to join the two triangles forming the rhombus-shaped DNA origami templates in the gold nanorods assemblies. The telomeric sequences folded into DNA G-quadruplexes when incubated with the azobenzene compound in the *trans* configuration. Upon exposing the assemblies to UV irradiation, the azobenzene molecules were converted into their *cis* isomer and dissociated from the quadruplexes, causing the telomeric DNA strands to stretch out, increasing the distance between the two gold nanorods. In contrast, upon visible light exposure, the azobenzene assemblies adopted the *trans* configuration, which stabilised the formation of G-quadruplexes in the telomere strands, decreasing the distance between the two. These proposed stimuli-responsive chiral plasmonic nanostructures had the ability to not only undergo conformational changes upon external stimuli but also produce CD responses in near infra-red (NIR) wavelengths, rendering them good candidates for optical reporting of crucial biological signals.

Recently, Samai and colleagues reported the assembly and optical characterisation of light responsive gold nanoparticles dimers linked by hairpin DNA modified with azobenzene molecules.^152^

**Fig. 18:**
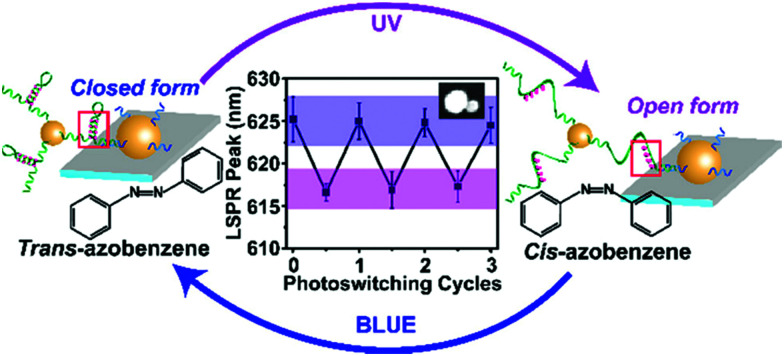
Schematic illustration of the light-responsive dimer assembly switching from the closed form to the open form and *vice versa* upon UV and blue light irradiation. The reversible change in the scattering spectra of approximately 100 dimers was achieved by numerous azobenzene photo-switching cycles. Reprinted with permission from *J. Phys. Chem. C* 2018, **122**(25), 13363–13370, Copyright© 2017 American Chemical Society.^152^

Exposure to UV light led to the denaturation of the hairpin, resulting in dimer extension and subsequent blue shift in the scattering spectra, while the process could be reversed by irradiation with blue light (Fig. 18). The plasmon peak shifts of approximately 100 individual dimer structures were analysed and it was found that the average interparticle distance for the *cis* azobenzene configuration (open state) was 17.7 ± 1.5 nm while for the *trans* azobenzene configuration (closed state) it was 14.3 ± 1.7 nm. These values were also confirmed by finite-difference time-domain electrodynamic simulations and were found to be in good agreement with the experimental results. Future studies based on this work, might look into further increasing the change in interparticle coupling by using dsDNA and refining the active photo-responsive sequence ratio to the length ratio of inactive “linking” DNA sequence with the aim of producing larger photo-reversible plasmon shifts.

Dai and Lo reported the successful self-assembly of a DNA nanotube structure with the ability to reversibly respond to visible and UV light irradiation.^153^ The fabrication of a light-responsive DNA nanotube was achieved by covalently incorporating three azobenzene moieties in a single DNA building strand. DNA hairpin structures were also incorporated into the DNA nanotube structure. Following visible and UV irradiation, photoisomerisation of azobenzene moieties occurred between the nonplanar *cis* and planar *trans* configurations resulting into the opening and closing of the cavities along the nanotubes. This further prompted the reversible change between the bent and linear nanotubes. Subsequently, 5 nm gold nanoparticles were longitudinally positioned on the 3D light-responsive DNA nanotube system providing orientational control over the gold nanoparticles as well as the distances between adjacent gold nanoparticles. The average interparticle distance altered from 8.5 ± 1.3 nm (in the closed state) to a bimodal distribution with values of respectively 8.39 ± 1.64 nm and 16 ± 4.3 nm (in the open state), and then reverted back to 8.35 ± 0.87 nm (in the closed state).

**Fig. 19:**
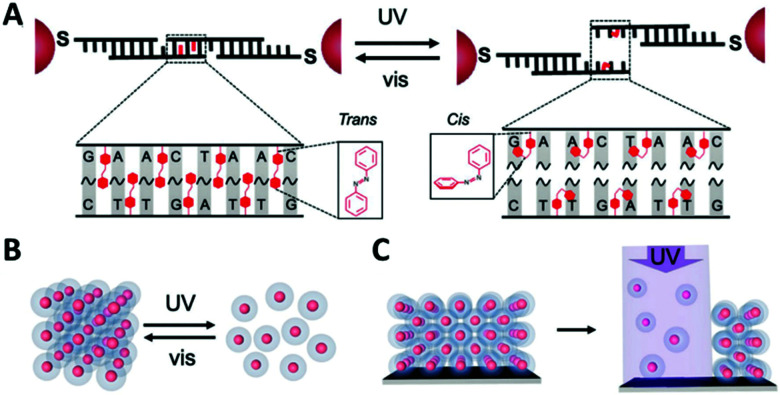
(A) Schematic illustration of the incorporation of the azobenzene moieties within the DNA sticky ends. (B) Assembly and disassembly of the Au superlattices under UV and blue light irradiation under isothermal conditions. (C) Disassembly of thin film of nanoparticles from Au superlattices, triggered by irradiation with UV light. Reproduced with permission from *Adv. Mater.*, 2020, **32**, 1906600, © 2020 WILEY-VCH Verlag GmbH & Co. KGaA, Weinheim.^154^

Light-responsive extended 3D networks (superlattices) were reported by the Mirkin group.^154^ The superlattices consisted of 10–30 nm gold nanoparticles assembled *via* complementary, azobenzene-modified DNA strands. The DNA linkers contained seven staggered azobenzene moieties positioned at the sticky ends of the strands. Owing to the azobenzene presence in the linker strands, the assembly or dissociation of the gold nanoparticle superlattices occurred as a result of the *trans* and *cis* configurational changes, respectively (Fig. 19). Additionally, UV light could trigger the selective removal of nanoparticles from both 3D crystals and bi-dimensional films. This study was also the first to demonstrate that through photo-patterning techniques, gold nanoparticle ordered arrays can be used to customise surfaces. These results provide a proof of concept that nanoparticle superlattices can be used to construct functional materials that could potentially be combined with current optical circuitry or microelectronics.

A different strategy for the photo-responsive reversible assembly of gold nanoparticles was demonstrated by Kanayama and co-workers.^155^ In their study, they functionalised dsDNA with an azobenzene derivative with a d-threoninol linker for the reversible assembly of gold nanoparticles *via* end-to-end stacking between blunt-ended DNA duplexes. This interaction did not involve hybridisation between complementary ssDNA, but originated from the π–π stacking between terminal nucleobases coming in close proximity under the appropriate ionic strength conditions. Gold nanoparticles functionalised with ssDNA assembled upon addition of the fully complementary ssDNA involving the *trans*-azobenzene d-threoninol moiety (at 1 M NaCl concentration). Interestingly, no assembly was observed when the modified oligonucleotide was added to a solution of ssDNA functionalised gold nanoparticles with a terminal T–T mismatch, confirming that in these conditions the assembly of gold nanoparticles was induced by the stacking interaction between the terminal A–T base pairs. UV light irradiation (*λ* = 350 nm) induced *trans*-to-*cis* isomerisation of the azobenzene moiety, unpairing the terminal bases and disrupting the end-to-end stacking, resulting in a gradual colour change from light purple to red, demonstrating the re-dispersion of the gold nanoparticles Subsequently, upon visible light irradiation (*λ* = 450 nm), *cis*-to-*trans* isomerisation of the azobenzene moiety was induced, leading to the re-pairing of the terminal bases and assembly of gold nanoparticles. The reversibility and stability of the system was proved up to 20 cycles. The versatility and the simplicity of this approach as well as the incorporation of only a single photo-responsive moiety, render it applicable for a variety of DNA nanostructures involving DNA origami, DNA tiles and DNA-based nanostructures.

As described above, a significant number of studies have utilised azobenzene modified DNA for nanoparticle assembly, (with particular focus on metallic nanoparticles); and new classes of photoswitches (*e.g.* arylazopyrazoles, spyropyrans) are emerging for application in photocontrollable DNA assemblies.^156–160^ The main advantage of the studies reviewed in this section is the universal applicability of the approach, making azobenzene-modified DNA a key effector in the assembly of various types of nanoparticles. Nanoparticle assemblies produced by azobenzene-modified DNA have diverse applications in the fabrication of optically interesting structures due to the facile *trans*-to-*cis* isomerisation of azobenzene upon visible and blue light irradiation which is useful for manufacturing multi-responsive nanostructures in optics, sensing and drug delivery.

## 4. Mechanically interlocked DNA nanostructures

**Fig. 20:**
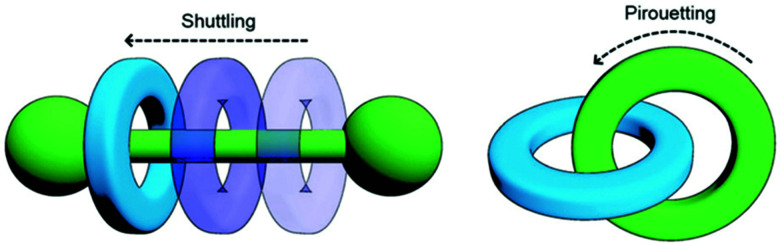
Changes in co-conformation of rotaxanes (left) and catenanes (right) as a result of shuttling or pirouetting. Reproduced with permission from N. Hoyas Pérez and J. E. M. Lewis, *Org. Biomol. Chem.*, 2020, **18**, 6757, Published by The Royal Society of Chemistry.^161^

Mechanically-interlocked molecular architectures (MIMAs) are connections of molecules that are linked together as a result of their topology rather than through chemical bonds. They cannot be separated without breaking a covalent bond. MIMAs are often used in molecular machines as the individual components can slide (shuttling) or rotate (pirouetting) relative to each other but cannot come apart. Common examples of MIMAs include rotaxanes and catenanes (Fig. 20), but many others exist such as molecular knots and Borromean rings.^161^

A rotaxane configuration consists of a macrocycle (ring) which is threaded onto a dumbbell shaped molecule. The name rotaxane derives from the Latin words rota (wheel) and axis (axle) and the dumbbell structure is referred to as the axle. The ends of the axle (often called stoppers) are large enough to prevent the macrocycle from unthreading. In comparison, catenanes consist of two or more mechanically interlocked macrocycles in a chain (catena-like configuration). Both rotaxanes and catananes are present in nature. For example, a variety of peptides have rotaxane substructure^162^ including microcin J25 (MccJ25),^163^ and catenated DNA forms during DNA replication,^164^ which was observed as early as 1967.^165,166^ Catenanes were first proposed in 1912 by Willstätter^167^ and the first synthetic reports for catenanes and rotaxanes date back to the 1960's.^168,169^ Whilst these early attempts were low yielding and inefficient, catenanes and rotaxanes can now be synthesised from a plethora of small discrete molecules and oligomers in high yield.^161^

This section will focus on the use of DNA catenanes and rotaxanes for the programmable self-assembly of nanoparticles. We will discuss their preparation and the chemical modifications that enable these constructions. DNA scaffolds enable the precise positioning of other species onto the MIMA as well as control over the length of dumbbells or size of macrocyclic and catenane rings. Moreover, DNA allows the construction of MIMAs that can respond to external stimuli, which is of benefit when constructing molecular machines. DNA structures can be designed so that they change in response to pH (i-motifs), triplexes, presence of cations including K^+^ (G-quadruplex) or divalent metal cations, and temperature. The addition of external nucleic acid strands to DNA nanostructures can trigger a change in conformation which in the case of DNA MIMAs can lead to shutting or pirouetting of the components in a controlled manner.

### 4.1 DNA rotaxanes

**Fig. 21:**
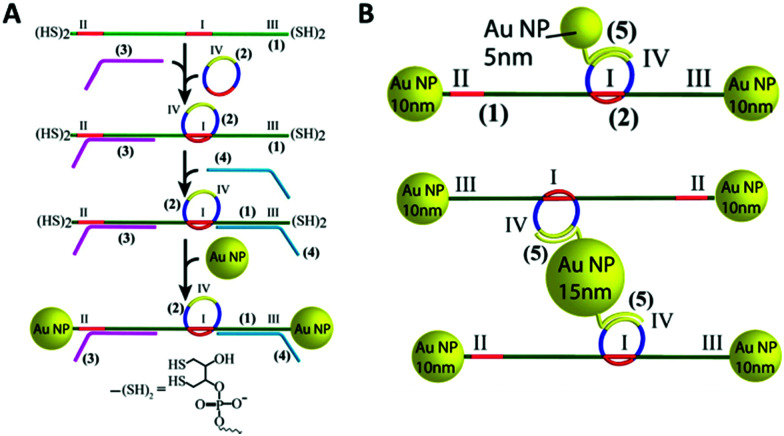
(A) Stepwise synthesis of gold nanoparticle stoppered DNA rotaxanes. In this design the DNA axle (1) and DNA ring (2) have a complementary region shown in red. After hybridisation of the axle (1), ring (2), and tether strand (3), a second tether strand (4) is hybridised to the axle. The resulting four-component nanoconstruct was then reacted with an excess of gold nanoparticles, installing the stoppers, before the tethers were removed. The DNA axle was chemically modified with the dithiol shown in the figure to allow functionalisation with the gold nanoparticles. (B) Different gold nanoparticle-rotaxane combinations can be accessed using this approach. Reprinted with permission *Nano Lett.* 2013, **13**(12), 6275–6280, Copyright© 2013 American Chemical Society.^171^

In 2010, the Famulok group reported the first rotaxane synthesised from DNA.^170^ Shortly after in 2013, a DNA rotaxane for nanoparticles assembly was published. In that work, a DNA ring was threaded onto a DNA axle that had two 10 nm gold nanoparticles as stoppers.^171^ This design required the use of a DNA axle that was chemically modified with dithiols at the 5′ and 3′ ends. The assembly of the construct is shown in Fig. 21. Short DNA strands functionalised with fluorescent dyes or different sized gold nanoparticles could then be hybridised to the DNA ring. The authors then demonstrated that the functionalised ring could be moved up and down the axle in a controlled manner by the addition of DNA strands that hybridise to the axle.

Gold nanoparticles have also been anchored on rotaxanes prepared using DNA origami.^172^ Unlike the design discussed in the previous example, the origami rotaxanes were larger (DNA origami rotaxanes were prepared with axle lengths of up to 355 nm) and structurally rigid. The authors postulated that transport of the ring could be controlled using plasmonic thermal cycling or optical switching. Due to the high programmability of origami structures, both the ring and the axle were customised with nanoparticles at precise locations and with defined stoichiometries. This system has the additional ability to potentially transport the components over the micrometre scale. Given these features, it is plausible that such designs will find application in programmable assembly lines, light driven plasmonic nanosystems, and sequential DNA-templated synthesis.

### 4.2. DNA catenanes

DNA catenanes have received increased research interest with a focus on the engineering of unique assemblies. To date, two-,^173^ three-,^174^ five-^175^ and seven-ring^176^ DNA catenanes have been synthesised. Recent developments regarding their synthesis and functions are reviewed by Lu and colleagues in their dedicated review.^177^ Here, we will focus only on the application of catenanes for the programmable self-assembly of nanoparticles.

**Fig. 22:**
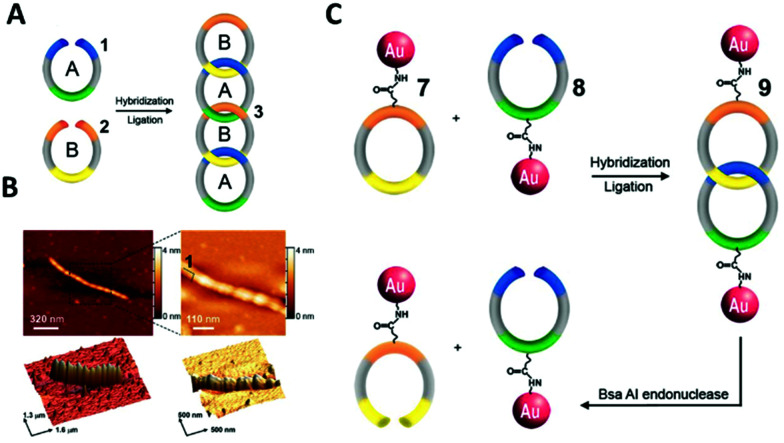
(A) Scheme showing the formation of the DNA polycatenated ladder. (B) Corresponding atomic force microscopy (AFM) of the resulting polycatenane ladder. (C) Representative scheme of the gold nanoparticles dimers from the interlocked A and B monomers. Reprinted from Y. Weizmann *et al.*, *Proc. Natl. Acad. Sci. U. S. A.*, 2008, **105**, 5289–5294., Copyright © (2008) National Academy of Sciences, USA.^178^

In 2008, Weizmann and co-workers developed a modular polycatenated DNA scaffold that could be used to program the assembly of gold nanoparticles, proteins, and several fluorescent dyes with precise spatial orientation.^178^ In this approach two linear ssDNA monomers were designed so that they could be hybridised together and treated with a ligase forming interlocked rings. The ligation of the ssDNA monomers’ ends generated a ladder comprised of alternating DNA rings as shown in Fig. 22. Gold nanoparticles-labelled ssDNA monomers were used to prove that functionalisation of the ssDNA monomers with large moieties did not hinder the catenation process. The DNA components of the gold nanoparticles-labelled monomers were synthesised with an internal amino-thymidine modification (presumably Amino C6 dT; however, details are not given), which was reacted with 14 nm mono-sulfo-*N*-hydroxy succinimide (NHS) gold nanoparticles forming an amide bond between the DNA and gold nanoparticles. The gold nanoparticle-labelled ssDNA monomers were designed so that two ringed catenanes formed, effectively pairing the gold nanoparticles, which was confirmed using TEM. Subsequent treatment with a restriction enzyme (BsaAI) cleaved the hybridised DNA, which resulted in the dissociation of the majority of gold nanoparticles from the assembly, suggesting the break of the catenane. Although this study revealed that this system can be utilised for the self-assembly of gold nanoparticles and their subsequent enzymatic separation, higher order gold nanoparticles functionalised catenanes were not prepared. It is likely that this versatile approach can be employed to generate elaborate DNA catenane templates which, in turn, can be utilised in the hierarchical assembly of nanoparticles with well-defined spatial orientation. Potential applications of such systems could include new plasmonic devices and sensors.

**Fig. 23:**
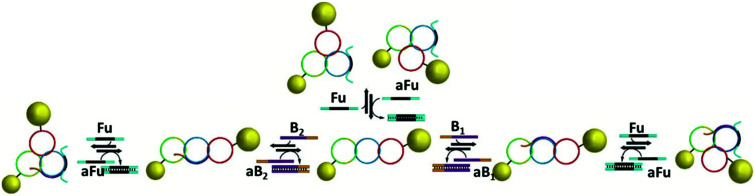
Schematic representation of the assembled 5 nm and 10 nm gold nanoparticles and how the configuration and corresponding interparticle distances can be controlled using different fuel and anti-fuel strands (Fu and aFu) or blocker strands (B or aB). Reprinted with permission from *Nat. Commun.***4**, 2000 (2013), Copyright © 2000.^179^

DNA catenanes have been used to prepare DNA-nanoparticle assemblies that allow the controlled switching between different configurations of gold nanoparticles, changing their interparticle distance. In 2013, Elbaz and colleagues reported a gold nanoparticles functionalised three-ring catenane molecular assembly that was able to switch configuration in response to the addition of various fuel and blocker ssDNAs as shown in Fig. 23.^179^ This approach could be used for the programmable production of reconfigurable gold nanoparticles or gold nanoparticles/fluorophore assemblies with controllable plasmonic properties.

**Fig. 24:**
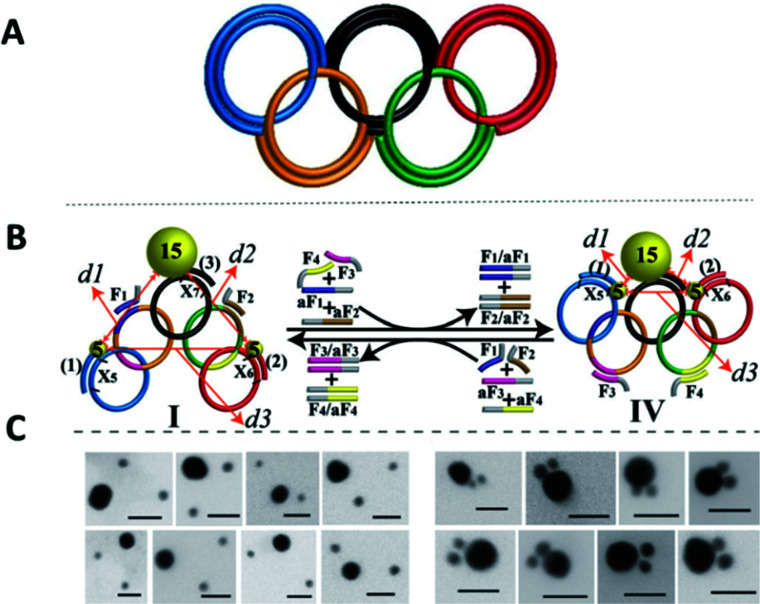
(A) Graphical scheme of the interlocked DNA olympiadane system. (B) Scheme for the assembly and reconfiguration of two 5 nm and one 15 nm gold nanoparticles. (C) Corresponding STEM images. All scale bars at 20 nm. Adapted from Lu *et al.*, *Angew. Chem.*, *Int. Ed.*, 2014, **53**, 7499–7503, copyright © 2014 WILEY-VCH Verlag GmbH & Co. KGaA, Weinheim.^175^

The Willner group reported the use of a DNA olympiadane (an interlocked five-ring catenane that resembles the Olympic rings) as a scaffold for different sized gold nanoparticles (Fig. 24). The olympiadane scaffold functioned as a molecular machine that alters the structure and position of the gold nanoparticles when the appropriate ssDNA fuel strands were added, (Fig. 24B).^175^ Two nucleic acids (marked as 1 and 2 in Fig. 24B) functionalised with 5 nm gold nanoparticles were hybridised to their complementary region in the red and blue rings. Similarly, a 15 nm gold nanoparticles attached to nucleic acid 3 was hybridised to the black ring. The assembled molecular machine was in configuration state I, and scanning transmission emission microscopy (STEM) analysis revealed that the gold nanoparticles were separated by the following approximate distances: *d*1 ≈ 14.5 nm, *d*2 ≈ 25 nm and *d*3 ≈ 32 nm as indicated by the orange arrows in Fig. 24B. Addition of ssDNA fuel strands F_3_ and F_4_ and antifuel strands aF_1_ and aF_2_ led to the reconfiguration of the machine, resulting in an olympiadane configuration (state IV). STEM analysis revealed that the gold nanoparticle distance had changed (*d*1 ≈ 0.75 nm, *d*2 ≈ 1.5 nm and *d*3 ≈ 2 nm) confirming that the system was in configuration state IV. Further treatment of state IV with fuel strands F_1_ and F_2_ and antifuel strands aF_3_ and aF_4_ returned the system to state I (Fig. 24C). This study demonstrated that the five-ring DNA catenane system effectively acts as a scaffold onto which nanoparticles can be assembled and subsequently reconfigured by external stimulation using the appropriate DNA strands. The exact positioning of nanoparticles and the control of their topography in combination with the versatility of DNA structures that can be made, suggest applications as memory systems and molecular logic operations devices.

**Fig. 25:**
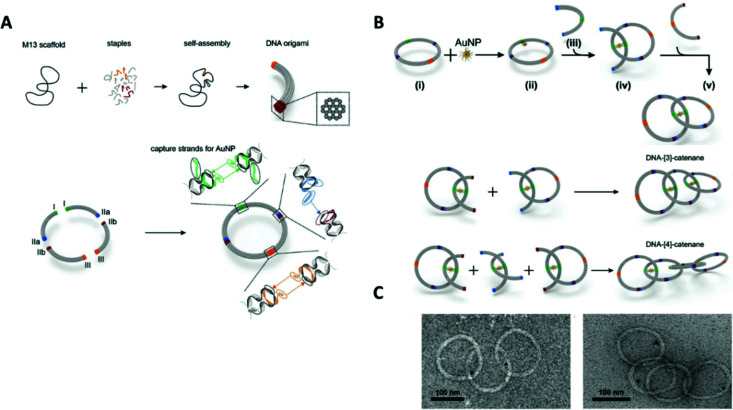
(A) Assembly of the origami ring through the bending of a M13 scaffold to form a curved monomer, followed by the junction of four monomer arcs. (B) A DNA-functionalised gold nanoparticles is used to mediate the formation of 2-, 3- and 4-ring catenanes. (C) TEM images of a DNA-[3] and a DNA-[4] catenane entwined by gold nanoparticles. Reproduced with permission from Peil *et al.*, *Nat. Nanotechnol.*, 2010, **5**, 712–717. © 2020 The Authors. Published by WILEY-VCH Verlag GmbH & Co. KGaA, Weinheim.^181^

Whilst DNA catenanes are topologically well-defined, they are also rather floppy and flexible as they are typically constructed from ss- or dsDNA. This can be a desirable or disadvantageous feature depending on the application. By comparison, interlocked structures that involve DNA origami are much more rigid. Yan and colleagues were the first to prepare DNA catenanes from DNA origami.^180^ In 2020, Peil and colleagues reported the use of gold nanoparticles as a template for the hierarchical assembly of four DNA origami catenanes.^181^ One ring was constructed from four DNA origami monomers as shown in Fig. 25. Each ring was approximately ∼120 nm in diameter with four different junctions (I, IIa, IIb and III). When junction I is assembled, four capture strands were available and could be used to anchor the templating gold nanoparticles (Fig. 25B). Two halves of the ring could then be entwined using the gold nanoparticles which were then hybridised with the other halves of the DNA origami ring forming the catenane structure. This design enabled the formation of 2-, 3- and 4-ring catenane structures using the gold nanoparticles as mediators (TEM micrographs shown in Fig. 25C). Whilst this approach does not focus on the assembly of gold nanoparticles but rather their use as a template for the assembly of the DNA origami catenane rings, it is a very promising strategy for the hierarchical assembly of nanoparticles with the advantage of producing much more rigid assemblies.

## 5. Nucleic acid backbone modifications

A variety of nucleic acid backbone modifications have been developed for therapeutic and diagnostic applications, and are now being adopted by researchers in the field of nanoparticle assembly.^182,183^ These modifications include changes to the furanose ring in DNA and substitution of the phosphodiester linkages with alternative chemistries. In general, the aim of these is to: (i) improve the stability of the oligonucleotide in biological or other settings; (ii) increase the affinity for complementary nucleic acid strands whilst maintaining exquisite selectivity; and (iii) have low toxicity. In this section we will focus on three different backbone modifications Locked Nucleic Acids (LNAs),^184–186^ Peptide Nucleic Acids (PNAs),^187^ boranophosphate DNA (BP-DNA),^188^ and phosphorothioate (PS-DNA), describing the properties of each and giving examples of their application in nanoparticles assembly.

### 5.1. Locked nucleic acids

**Fig. 26:**
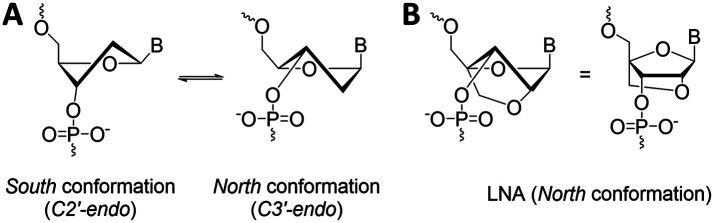
Structure of LNAs. (A) The equilibrium between the C2′-endo and C3′-endo sugar conformations present in nucleic acids. (B) The methylene bridge in LNA locks the sugar in the North conformation. B = nucleobase (A, T, 5-methyl C or G).

Locked nucleic acids (LNAs) are nucleotide analogues that contain a methylene bridge linking the 2′ oxygen and the 4′ carbon of the furanose ring. This bridging carbon freezes the sugar in the North (3′ *endo*) conformation, which pre-organizes the oligonucleotide backbone ready for binding to complementary RNA and DNA strands (Fig. 26).^189^ LNAs have strong binding affinity and selectivity for complementary DNA and RNA strands. The greater duplex stability relative to unmodified DNA is reflected in UV melting studies where the addition of a single LNA modification to DNA sequences typically increases the binding affinity towards RNA by around 7 to 8 °C and binding to DNA by around 5 °C.^190^ Moreover, LNA modifications can be incorporated into oligonucleotides by standard solid-phase oligonucleotide synthesis allowing the number of LNA modifications within an oligonucleotide to be varied to tune the hybridisation properties. As a result, LNA modifications have had a major impact in nucleic acid research and biotechnology,^186,189,191^ and they are now commercially available. LNAs have found applications in material science, molecular diagnostics and biotechnology, while addressing challenges such as target binding selectivity as well as contributing to enzymatic and chemical stability.^192,193^ The synthesis of LNA and a wide range of LNA applications and their use as therapeutics has been covered in multiple reviews.^186,192–195^

Despite the obvious beneficial properties of LNA, the full potential of LNAs in the programmable assembly of nanoparticles remains largely underexplored. In the following section we will describe the current state of the art.

The beneficial properties of LNA in nanoparticle assemblies were first reported in 2007 by McKenzie and colleagues who demonstrated that nanoconjugate probes built from LNA modified gold nanoparticles possessed several advantages in comparison to those that used DNA modified gold nanoparticles.^196^ Their simple nanoconjugate probe design used two different nucleic acid modified gold nanoparticles, which assembled in a tail-to-tail fashion against target DNA, forming a 3D network of gold nanoparticles. Due to a synergistic effect occurring between the LNA and gold nanoparticles, these conjugates demonstrated greater stability and binding affinity when compared to DNA functionalised gold nanoparticles. Using a similar principle, the same group developed probes for the detection of dsDNA,^197^ avoiding the need to isolate DNA in its single stranded state for detection. In this example, gold nanoparticles were functionalised with LNA modified triplex-forming oligonucleotides (TFOs). TFOs bind to the major grove of a DNA duplex in a sequence specific manner but require protonation of the deoxycytidine base to form stable CGC triplets. The use of LNA was integral to the probe design as it allowed the probes to function under neutral rather than acidic conditions. Importantly, the ratio of LNA to DNA nucleotides used in the oligonucleotides was 3 : 7, as this is known to give the best thermal stabilities whereas fully modified LNA strands are not capable of triplex formation.^198^ In the presence of the target dsDNA, a colour change from pink to blue was observed.

**Fig. 27:**
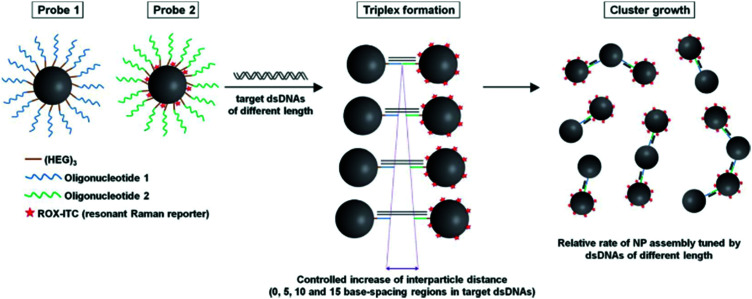
Initially, the head-to-head triplex assembly of silver nanoparticles using LNA TFOs occurred. Following the formation of the initial dimer, the nanoparticles assembly resumed by the formation of larger nanoparticle clusters, with the growth rate determined by the length of the DNA. Reproduced from Guerrini *et al.*, *Chem. Sci.*, 2012, **3**, 2262–2269 with permission from The Royal Society of Chemistry.^199^

In 2012, the Graham group applied their TFO approach to silver nanoparticles to control the interparticle distance in the assemblies, which enabled them to investigate the correlation between plasmonic and SERS properties.^199^ Understanding the link between these two properties is significant for designing optically-active nanostructures for SERS applications. To achieve this, silver nanoparticles (48 ± 5 nm in diameter) were functionalised with dye-labelled LNA sequences *via* a thiol modification. The LNA sequences used were TFOs, and nanoparticle assembly was induced by the addition of complementary dsDNA targets (Fig. 27). The central region of the dsDNA targets was not complementary to the silver nanoparticles/TFO probes and comprised of 0, 5, 10 or 15 base pairs, which accurately controlled the interparticle distances. The dsDNA targets used were highly rigid structures and enabled the direct determination of the SERS enhancement responses and G gap-plasmon produced by the nanoparticle assemblies as a result of the carefully controlled interparticle distances. Interestingly, the shorter the dsDNA target used, the faster aggregation occurred.

The use of LNAs for the assembly and disassembly of polystyrene nanoparticles, using short duplexes that are exact matches or contain one centre mismatch, was explored in a study conducted by Eze and Milam.^200^ Initially, primary hybridisation and competitive displacement activities involving two matched or mismatched DNA–DNA, LNA–LNA, and LNA–DNA strands were quantified as a function of sequence complementarity and length. Programmable colloidal assembly and disassembly of polystyrene nanoparticles was achieved by successfully tuning and optimising the hybridisation affinity, sequence length, duplex concentration and number of LNA residues found in the probe and target strands. Satellite structures were produced for the assembly and disassembly experiments by utilising individual non-fluorescent microspheres (1 μm in diameter) surrounded by red fluorescent polystyrene nanoparticles (200 nm in diameter). Although very low duplex density was observed regarding satellite assembly, the resulting structures were well-dispersed and stable enough to withstand multiple washing steps (with only few dimer or trimer structures present). Disassembly was induced by the addition of longer, perfectly complementary DNA or LNA target strands into the assembled satellite suspension. This resulted in competitive displacement of the weaker original strands (used for the satellite structure assembly), releasing the red fluorescent polystyrene nanoparticles from the surface of the microspheres. This study demonstrated a comprehensive strategy for the LNA-mediated assembly and disassembly of satellite colloidal structures that are protected from nuclease degradation by the use of LNA, allowing physiological applications that may not otherwise be achievable using DNA.

**Fig. 28:**
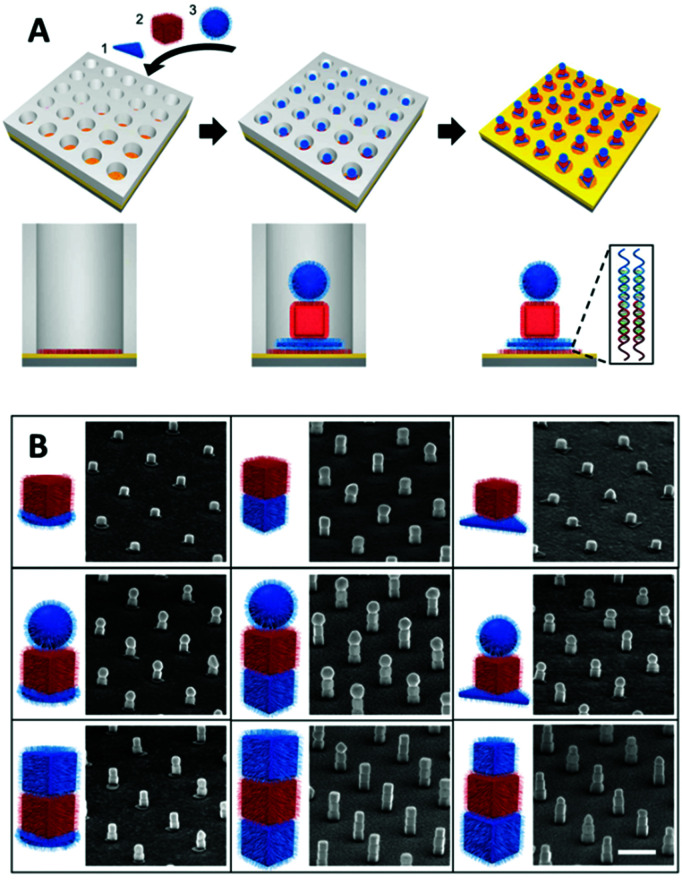
(A) Polymer pore template for the layer-by-layer DNA-directed assembly of various shapes and sizes of colloidal gold nanoparticles, resulting in well-oriented gold superlattices. In the first step, 1D pores are fabricated in a PMMA-coated gold substrate using lithography, and the Au surface at the bottom of each pore is functionalised with oligonucleotides. In the second stage, DNA-functionalised colloidal nanoparticles are assembled in a layer-by-layer manner by designing each layer of nanoparticles to have a terminal DNA sequence complementary to that of the previous layer. Finally, the porous PMMA template is removed to generate gold nanoparticles superlattices with 2D periodicity that are composed of gold nanoparticles architectures. Bottom images depict cross-sectional views of a single pore. (B) Schematic illustrations and corresponding SEM images of the assembly of well-oriented superlattices, comprised of two- and three-layer nanoparticles structures. Adapted from Lin *et al.*, *Science*, **359**(6376), 669–672, DOI: 10.1126/science.aaq0591, Copyright © 2018 The Authors.^201^

In 2018 Mirkin′s group used a polymer pore template for the layer-by-layer DNA-directed assembly of colloidal gold nanoparticles into various shapes and sizes, resulting in well-oriented superlattices (Fig. 28).^201^ Whilst numerous examples of DNA-mediated nanoparticle assembly in different crystalline structures exist,^202^ this study was unique as it enabled the assembly of discrete superlattices composed of multicomponent nanoparticle architectures with controlled orientation, arrangement, and spacing. In their approach, electron beam lithography is first used to cut pores in a poly(methylmethacrylate) (PMMA) coated gold surface and the exposed gold surface in the submicron sized pores is then functionalised with propylthiol modified DNA. Complementary oligonucleotides were hybridised to these strands resulting in a single layer of rigid duplexes with a short single stranded region or ‘sticky end’ facing out of the pore. This ‘sticky end’ was designed to hybridise to DNA functionalised gold nanoparticles. The replacement of three adenine nucleotides in the sticky-end with LNA equivalents was key to this strategy. Compared to the DNA sticky ends, the LNA modifications increased the duplex stability with the target gold nanoparticle DNA strands, raising the melting temperature by 9 °C, whilst also decreasing the melting temperature of the off-target interactions by 1 °C. This improved the temperature window addressing the challenges experienced with the DNA sticky ends and enabled the synthesis of highly ordered superlattices involving up to three layers of structures. These new nucleic acid designs have the potential to extend responsiveness to chemical and biological signals, as well as light, providing access to improved optical properties.

### 5.2. Peptide nucleic acids

**Fig. 29:**
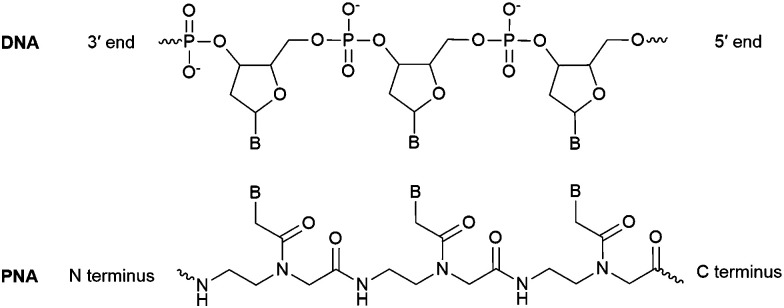
Chemical structure of a PNA:DNA duplex. In PNA the regular phosphodiester backbone of a DNA strand is replaced by 2-aminoethyl glycine linkages. B = nucleobase (A, T, C or G).

Peptide nucleic acids (PNAs) were first reported in 1991 by Nielsen *et al.*^187^ In PNAs, the sugar-phosphate backbone present in DNA is replaced with a repeating *N*-(2-aminoethyl)glycine (AEG) backbone, which renders the PNAs neutral in charge (Fig. 29).^187,203,204^ As a result of their neutral nature, PNAs possess (i) strong binding affinity to their complementary DNA or RNA strands regardless of salt concentration; (ii) poor water solubility (when compared to DNA); and (iii) high stability in biological systems as a result of degradation resistance to protease and nuclease activity.^190,205–208^ The biological stability and high target affinity of PNAs have led to their broad applicability in detection and diagnosis,^209–214^ as antisense and antigene therapy candidates,^215–219^ and as biosensors.^220–223^

PNAs are achiral and can be synthesised without having to follow a stereoselective pathway.^203,205^ Typically, PNA oligomers are synthesised by solid phase chemistry that is analogous to peptides synthesis.^205,224^

Functionalisation of nanoparticles with PNAs, represent a very promising strategy for the fabrication of stable nanoparticles assemblies, independent of the local ionic strength. In 2003, Chakrabarti and Klibanov demonstrated that PNAs can be used to drive the self-assembly of gold nanoparticles.^225^ In detail, thiol modified PNA or ssDNA were functionalised on gold nanoparticles and they were assembled with complementary ssDNA or PNA gold nanoparticles to form various types of assemblies (PNA–PNA, PNA–DNA, DNA–DNA). A key advantage of the PNA–PNA assembly system was that stable hybridisation could be achieved using a recognition length of only few bases. Another feature of the PNA-based systems was that a single base mismatch was enough to prevent nanoparticles from assembling, whereas a partial assembly was still observed in the DNA–DNA system, suggesting that PNA-driven assemblies may be more selective in recognition of mismatches. This might represent an advantage over DNA driven assemblies in the development of sensors and diagnostics, where high selectivity is required. The size and the rate of assembly was also determined by the PNA amino acid composition. Considering these observations along with the ease of modifying the polyamide backbone by incorporating canonical amino acids during the synthesis, PNA constitutes a strong candidate for manipulating nanoparticle self-assembly.

The hybridisation and melting behaviour for PNA–DNA chimera coated gold nanoparticles was reported by Murphy *et al.*^226^ PNA–DNA chimeras consisted of seven DNA nucleotides linked to five PNA bases and a 5′ terminal thiol modification was used to tether the chimeras to the gold nanoparticles. The hybridisation and melting properties of complexes formed between these chimera–gold nanoparticles and DNA–gold nanoparticles were studied using UV-Vis melting, and it was observed that the PNA–DNA chimera melting profiles were sharper than the corresponding DNA–gold nanoparticles complexes.

**Fig. 30:**
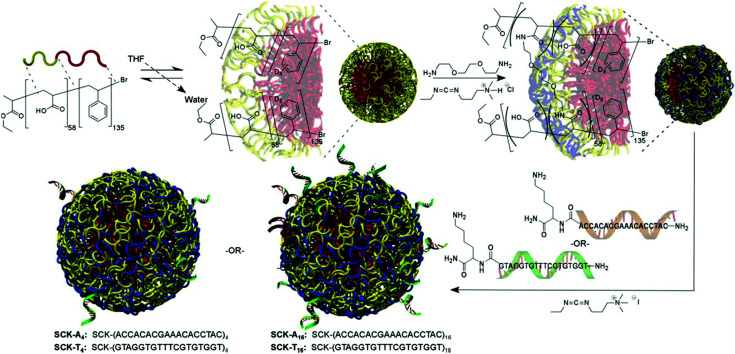
Schematic illustration of the assembly and subsequent PNA functionalisation of SCK nanoparticles. The introduction of water induced micellisation of the diblock copolymer of polystyrene(D_8_)-*b*-poly(acrylic acid), which was subsequently followed by the capture of the nanostructure through crosslinking. Carbodiimide coupling was then used for the conjugation of PNAs to the nanoparticles. Reproduced from Turner *et al.*, *Soft Matter*, 2005, **1**, 69–78, with permission from The Royal Society of Chemistry.^227^

PNA has been used in the assembly of non-metallic nanoparticles such as shell crosslinked Knedel-like (SCK) nanoparticles. SCK-nanoparticles consist of a hydrophobic core and a charged cross-linked shell of blocked copolymers.^227^ SCK-nanoparticles have the advantage of being chemically versatile while the core composition can involve crystalline, fluid-like or glassy materials. In addition, the shell can be negatively or positively charged. To direct the self-assembly of SCK nanoparticles, Turner and colleagues developed a method to conjugate PNA to the surface of SCK nanoparticles. In their experiments, the SCKs used were 18 ± 4 nm in size and consisted of hydrophobic, glassy polystyrene cores linked covalently to a hydrophilic, hydrogel-like poly(acrylic acid-*co*-acrylamide) shell. PNAs were prepared with a lysine residue at the C terminus, which provided a free primary amine that could be coupled to carboxylic acids present in the shell of the SCKs (Fig. 30). Subsequently, PNA–SCK nanoparticles were prepared and used as building blocks for the fabrication of hierarchical structures.

**Fig. 31:**
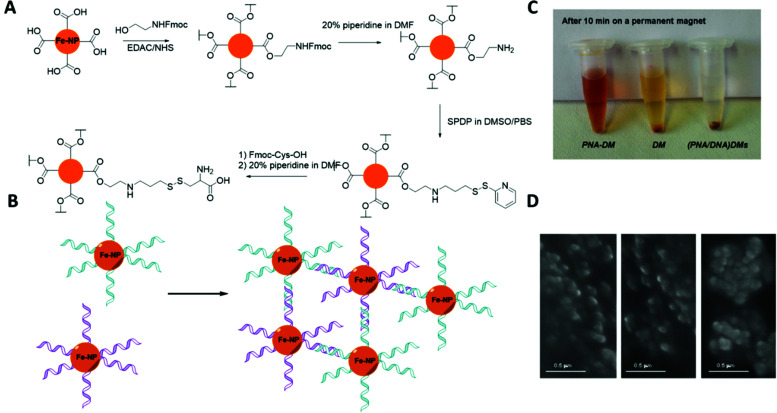
(A) Preparation route of the dextran magnetite nanoparticles with cysteine functionalisation. (B) Schematic illustration of the assembly of PNA and DNA functionalised magnetic nanoparticles. (C) Photograph of free nanoparticles and magnetic nanoparticles conjugates following a 10 min application of on a permanent magnet. (D) From left to right: Ultra high vacuum scanning electron microscopy (UHV-SEM) images for (i) DM assemblies; (ii) PNA-DM assemblies; and (iii) (PNA/DNA) DM assemblies. Adapted from *Mol. BioSyst.*, 2010, **6**, 553–561, with permission from The Royal Society of Chemistry.^228^

Milano and co-workers reported a new strategy for synthesising DNA- and PNA-conjugated biocompatible 130 nm dextran-magnetite particles (DMs) (Fig. 31).^228^ The functionalisation of the DMs with PNA or DNA is depicted in Fig. 31A. The PNA or DNA oligomers were introduced on the surface of the poly-functionalised DMs using 1-ethyl-3-(3-dimethylaminopropyl)carbodiimide/N-hydroxysuccinimide (EDAC/NHS) coupling. When the complementary DNA- and PNA-nanoconjugates were combined with each other (owning to the polyvalent nature of the nanoparticles), the formation of duplex structures occurred.

Anstaett and colleagues were the first to synthesise PNA-functionalised 40 nm gold nanoparticles stabilised by thiolated alkyl PEG carboxylate surfactants and assemble them on gold surfaces in the absence of ions or other surfactants.^229^ The authors highlighted the importance of ion free conditions for investigation of electrostatic phenomena and the benefit of the increased binding strength of PNA hybrids for sensing applications.

### 5.3. Borane phosphonate DNA

**Fig. 32:**
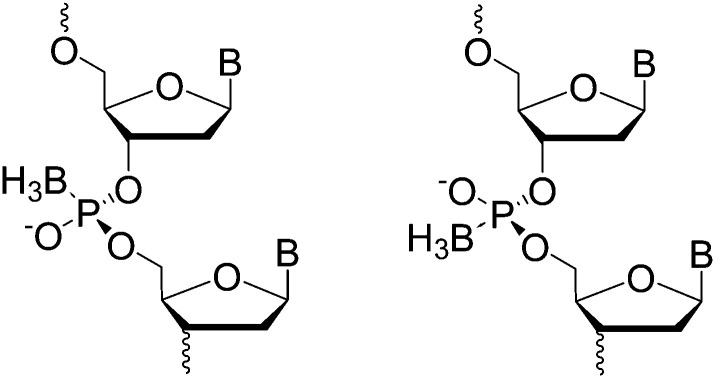
All S_p_ BP-DNA (left) and all R_p_ BP-DNA (right).

In borane phosphonate DNA (BP-DNA) an oxygen in the phosphodiester linkage of DNA is replaced with an electron deficient borane (BH_3_) (Fig. 32).^188,230^ BP-DNA is a promising class of nucleic acids that shows high levels of gene suppression,^231–233^ higher stability to nucleases than the commonly used phosphorothioate modification,^234,235^ and high lipophilicity due to the borane group. The increase in lipophilicity improves its cellular activity.^233^ The affinity of BP-DNA to RNA is dependent on stereochemistry. The boranophosphate linkage has P-diastereoisomerism with two different absolute configurations (S_p_ and R_p_) (Fig. 32). Early reports suggested that BP-DNA prepared using enzymes (which would be stereopure) shows a reduction in affinity for RNA compared to DNA, and BP-DNA synthesised as a diastereomeric mixture has much lower affinity towards its target.^234^

BP-DNA has the ability to reduce metal ions such as Pt(ii), Ag(i) and Au(iii) resulting in the production of the corresponding metallic nanoparticles, essentially enabling the DNA-induced spatial placement of nanoparticles on DNA nanostructures as well as morphology control.^236^

**Fig. 33:**
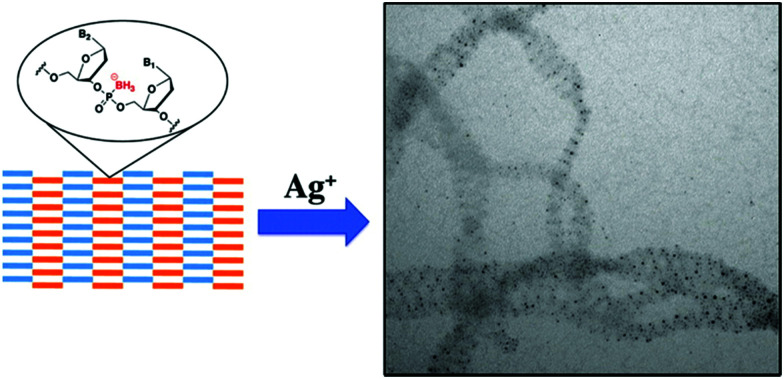
Schematic illustration of BP-DNA utilised for the assembly silver nanoparticles arrays. The orange squares represent DNA tiles that contain BP-DNA whereas the blue tiles consist of unmodified DNA. Upon exposure to silver salts silver nanoparticles form. These can be visualised using TEM as shown on the right. Reproduced from Roy *et al.*, *J. Am. Chem. Soc.*, 2013, **135**(16), 6234–6241, Copyright © 2013 American Chemical Society.^237^

In 2013 the Caruthers’ group reported a new chemical synthesis strategy that enabled them to prepare long and pure BP-DNA. This new strategy enabled the incorporation of BP-DNAs into a 2D DNA array made up of two tiles involving double crossover junctions.^237^ Only one of these tiles was programmed to include BP-DNA. Therefore, upon exposure of this DNA array to a silver salt, silver nanoparticles were selectively deposited onto the tiles in a pre-programmed arrangement (Fig. 33). Depositing metal onto intricate DNA assemblies is a particularly promising strategy for achieving metallic nanostructures which are smaller than what can currently be prepared using top-down lithographic methods. An added advantage to this method is the lack of extra synthetic and purifications steps. The concentration and the spatial distribution of the BP-DNAs can easily be altered, therefore offering significantly high control over the density and the size of the resulting metallic features.^237^

Two years later, an alternative strategy for silver nanoparticle assembly using BP-DNA was reported, this time using rolling circle amplification (RCA) to enzymatically generate the BP-DNA. In RCA, a cyclic DNA template is traversed many times by a DNA polymerase (typically phi29) generating long ssDNA, which is formed from repeating segments that are complementary to the cyclic template. By adding 5′-(α-P-borano) deoxy-nucleoside triphosphates (bdNTPs) to the amplification mixture the authors were able to generate micrometre sized material formed from BP-DNA. This material was then metallised by treatment with silver ions (Ag^+^) producing silver nanoparticles along the RCA backbone. Also, the authors varied the composition of deoxynucleotide triphosphates (dNTPs) and bdNTPs allowing them to control the proportion of phosphodiester and boranophosphonate linkages in the BP-DNA product.^238^ By varying these parameters, one could control the distribution of silver nanoparticles along the rolling circle product. RCA can be used to generate a wide variety of structures and materials and this approach could potentially unlock a new array of nanoparticle assemblies that could not be constructed using other methods described in the review.

**Fig. 34:**
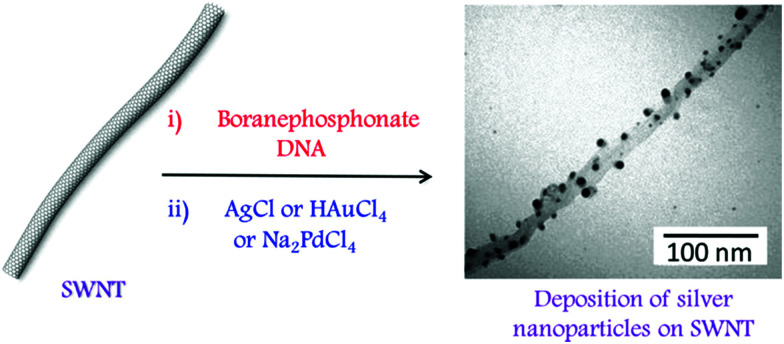
Schematic illustration of single-walled carbon nanotube and TEM image of silver nanoparticles assembly on BP-DNA functionalised single-walled carbon nanotubes. Reprinted from *Chem. Mater.* 2017, **29(**5), 2239–2245, Copyright © 2017 American Chemical Society.^239^

The Caruther′s group have also used chemically synthesised BP-DNA as a metallisation agent for single-walled carbon nanotubes (SWNTs). Here, the BP-DNA was first adsorbed onto the surface of SWNTs, before treating the SWNTs with silver, gold, or platinum salts. The boranophosphonate group subsequently induced the formation of the corresponding nanoparticles by the reduction of their ions without the presence of additional reducing agents (Fig. 34).^239^ Palladium nanoparticles anchored on the SWNTs were particularly good at catalysing the formation of C–C bonds *via* the Heck and Suzuki reactions, displaying improved activity compared to traditional Pd–C catalysts. Furthermore, the palladium nanoparticle-coated SWNTs were capable of reducing the redox dye 2,6-dichlorophenolindophenol (DCPIP), demonstrating the effective electron transfer properties of the metallised SWNTs. This BP-DNA-induced SWNTs metallisation strategy provided a promising approach for the fabrication of well-defined SWNTs utilised for nanoscale metal catalysis.

### 5.4. Phosphorothioate DNA

**Fig. 35:**
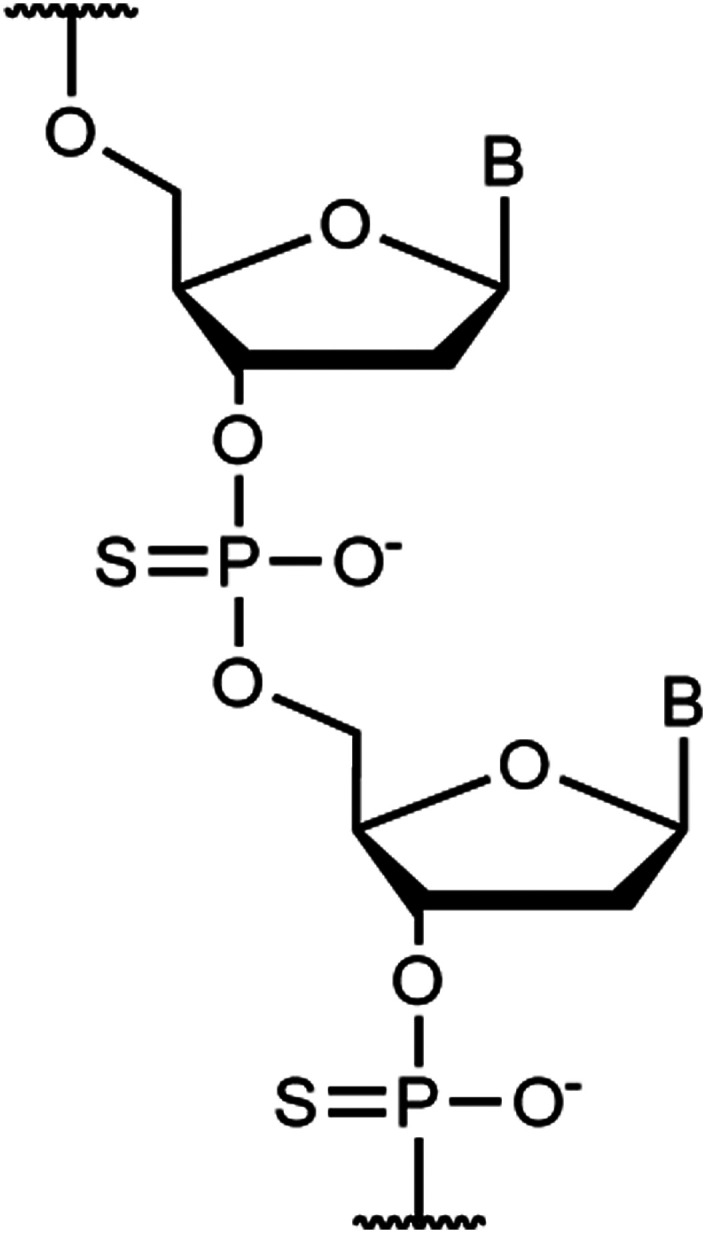
Chemical structure of a phosphorothioate oligonucleotides, where a sulfur atom. replaced a non-bridging oxygen on the phosphate backbone.

In phosphorothioate DNA (PS-DNA), a non-bridging oxygen in the phosphate backbone of an oligonucleotide is replaced by a sulfur atom (Fig. 35). This modification renders the inter-nucleotide linkage resistant to nuclease degradation, thus making PS–DNA suitable for *in vitro* and *in vivo* applications. The presence of multiple sulfur atoms along the backbone enhances the binding of nanoparticles on the DNA. Nanoparticles assembly mediated by PS–DNA can occur both *via* electrostatic interactions and covalent binding.^240^

In 2007, Lee and co-workers exploited this strategy by employing phosphorothioate DNA coupled with a short bifunctional molecular fastener for site-specific binding of gold nanoparticles.^241^ The authors demonstrated the precise positioning of nanoparticles along the PS DNA, both in solution and on surfaces. The same group reported later that by placing phosphorothioate modifications at several dsDNA positions they were able to assemble multiple proteins onto the backbone in precise locations. Additionally, they demonstrated the activity of conjugated proteins by the binding of biotinylated gold nanoparticles onto preassembled protein-dsDNA complexes, resulting in nanoparticle dimers and trimers with controlled distances.^242^

Following this work, in 2012, Wen and colleagues reported an easy way to prepare backbone-modified DNA by introducing a cyclic disulfide-containing phosphoramidite precursor during the automated oligonucleotide synthesis.^243^ This short cyclic disulfide group provided increased stability of the gold nanoparticle–DNA conjugate, and precise positioning of the nanoparticles. When gold nanoparticles were immobilized at the interior of the DNA sequence, the 5′ and 3′ sticky ends of ssDNA were still available for further functionalisation. The as-prepared DNA strands were employed in the fabrication of dimers, trimers, and tetramers.

Apart from gold nanoparticles, PS–DNA has been also utilised as a template for assembling other types of nanoparticles. Tikhomirov and co-workers^244^ reported the self-assembly of luminescent complexes using cadmium telluride nanocrystals capped with di-block DNA strands, consisting of a PS moiety to bind the QDs, and a phosphodiester fragment, for oligonucleotides hybridisation. Quantum dots containing from one to five DNA-based binding sites were synthesised and used as building blocks to create a variety of rationally designed assemblies, including linear and cross-shaped structures. Bimetallic satellite assemblies containing a silver nanoparticle core surrounded by gold nanoparticles were also fabricated by using oligonucleotides containing three, six or nine consecutive PS linkages, showing different assembling kinetics.^245^

The electrostatic assembly relies on the adsorption of the PS–DNA strands onto thiophilic particles, and it is superior in comparison with unmodified DNA. In 2005, Jiang and collaborators showed the possibility of constructing aggregates of gold nanoparticles with different 2D architectures by adjusting the length of PS oligo-DNA linkers.^246^ The architectures of the nanoparticles in the aggregates could be controlled by the different steric effects arising from the length of the PS DNA linkers employed. Another example of non-covalent assembly was presented by Shen *et al.*, who fabricated quantum dots with tuneable valences by employing a series of programmable DNA scaffolds. They demonstrated the use of these valence-engineered quantum dots to develop twelve types of topologically organized homo- and hetero-dimers of quantum dots and gold nanoparticles.^247^

LNAs, PNAs, BP-DNA and PS–DNA have specific characteristics that can be dominant in controlling nanoparticle self-assembly and the fabrication of larger nanostructures of interest. Some of their advantages are: (i) improved stability and mismatch sensitivity; (ii) the ability to tune the electrostatic and structural properties of nanoparticles assemblies; (iii) improved nuclease resistance over DNA; and (iv) the lack of further synthetic and purification steps. Chemically modified nucleic acids such as LNAs, PNAs, BP-DNAs and PS-DNAs have strong potential for further exploitation in nanoparticle self-assembly and certainly they will be further exploited for the fabrication of novel materials at the mesoscale.

## 6. Summary and outlook

DNA is a superior biopolymer that has been employed as a tool to assemble nanoparticles and larger nanostructures. Research in the field has vastly progressed in recent years and nowadays DNA has been utilised to arrange billions of nanoparticles in ways analogous to the configuration of atoms in crystal structures. Face-centred cubic, body-centred cubic and many other nanoparticle arrangements have been fabricated by controlling DNA density, length, base content and structure. DNA can be enriched with more functionalities by altering its chemistry, and the scope of this review was to discuss literature where DNA chemical modifications and small molecules have been used to manipulate nanoparticle organisation and the formation of larger mesoscale structures.

We have discussed general strategies that promote the ligation or disassembly of nanoparticles as summarised in Table 1. For example, when oligonucleotides modified with azide and alkyne groups come in close proximity, they react either spontaneously or with the help of a copper catalyst to form covalent chemical bonds. DNA ligation or disassembly between nanoparticles can also happen by using small DNA intercalating molecules (*e.g.* psoralen, azobenzene) or other photoactive molecules bound to oligonucleotides (*e.g.* carbazole derivatives), which are activated upon light irradiation. Using an external stimulus such as light to promote a photochemical reaction, and therefore to direct the ligation or disassembly of nanostructures, decouples the traditional dependence of DNA nanostructures on temperature and ionic conditions, enabling materials applications under a broader range of conditions. The modification of DNA by photoactive molecules is versatile, rendering this strategy attractive for the production of nanoparticle assemblies with potential applications for example, in the fields of optics, biosensing and drug delivery.^248,249^

**Table 1:** A table summarising the different chemical modifications for oligonucleotides and the key achievements

	Types of nanoparticle assemblies
Chemical modifications and intercalators	
Click chemistry DNA modifications (CuAAC)	• Gold nanoparticle aggregate assemblies^94^
	• Gold nanoparticle one-dimensional chain assemblies^95^
	• Upconversion aggregate assemblies^96^
	• Gold nanoparticle-biomolecules conjugate assemblies^97^

Click chemistry DNA modifications (SPAAC)	• Gold nanoparticle dimer assemblies^99,100,102^
	• Gold nanoparticle assemblies on graphene oxide nanosheets^101^
	• Gold nanoparticle dimer, trimer, cross-shaped and chain assemblies on DNA origami frames^104^

Vinyl DNA modifications	• Gold nanoparticle dimer, trimer, and tetramer assemblies^116^
	• Gold and Silver nanoparticle three-dimensional superlattice assemblies^117^
	• Gold nanoparticle two-dimensional assemblies^118^
	• Polystyrene nanoparticle surface assemblies^120^

Psoralen derivatives	• Gold nanoparticle wire assemblies on DNA template^129^
	• Gold nanoparticle dimer, trimer, and tetramer assemblies^131,132^
	• Gold nanoparticle three-dimensional superlattice assemblies^133^
	• Iron oxide nanoparticle assemblies on DNA template^130^

Azobenzene derivatives	• Gold nanoparticle dimer assemblies^152^
	• Gold nanorod dimer assemblies on DNA reconfigurable templates^149^
	• Gold nanoparticle trimer assemblies on DNA tetrahedra nanostructures^144^
	• Gold nanoparticle assemblies on three-dimensional DNA nanotubes^153^
	• Gold nanoparticle aggregate assemblies^145^
	• Gold nanoparticle three-dimensional superlattice assemblies^154^
	• Lipid unilamellar vesicles (LUVs) aggregate assemblies^150^

Mechanically interlocked DNA nanostructures	
DNA rotaxanes	• Gold nanoparticles assembled on DNA ring and DNA axle^177^
	• Gold nanoparticles assembled on DNA origami ring and DNA origami axle^172^

DNA catenanes	• Gold nanoparticles assembled on three DNA interlocked rings (various configurations)^178,179^
	• Gold nanoparticles assembled on five DNA interlocked rings (Olympiadane system)^175^
	• Gold nanoparticles assembled on three and four DNA origami interlocked rings^181^

Nucleic acid backbone modifications	
Locked nucleic acids (LNAs)	• Gold nanoparticle layer-by-layer superlattice assemblies^201^
	• Silver nanoparticle aggregate/cluster assemblies^199^
	• Polystyrene nanoparticle satellite assemblies^200^

Peptide nucleic acids (PNAs)	• Gold nanoparticle aggregate assemblies, including mixed PNA/DNA chimeras^225,226^
	• Gold nanoparticle array assemblies on template surface^229^
	• Shell crosslinked Knedel-like (SCK) nanoparticle hierarchical assemblies^227^
	• Dextran-magnetite particle (DMs) assemblies^228^

Borane phosphonate DNA (BP-DNA)	• Silver nanoparticle array assemblies on two-dimensional DNA arrays^237^
	• Silver nanoparticle assemblies on rolling circle amplification (RCA) DNA template^238^
	• Palladium, silver and gold nanoparticle assemblies on single-walled carbon nanotubes^239^

Phosphhorothioate (PS-DNA)	• Gold nanoparticle dimer, trimer, and tetramer assemblies^241,246^
	• Quantum dots assemblies^247^
	• Bimetallic silver and gold assemblies^245^

Versatile DNA structures such as rotaxanes and catenanes have enabled the formation of reconfigurable nanostructures. Information is encoded in the design of the oligonucleotides and enables the controlled rotation of DNAs within nanoparticle assemblies. As a result, the motion of nanoparticles (*e.g.* plasmonic, fluorescent or magnetic) can be independently controlled within nanostructures and therefore the collective properties of nanostructures can be tuned by manipulation of the interparticle distances. The production of reconfigurable nanoparticle assemblies *via* DNA catenanes and rotaxanes with well-defined spatial orientation, represents a promising strategy for the construction of memory devices as well as possible catalysis and sensing applications.^177^

The stability, rigidity and valency of nanostructures can also be tuned by chemical modifications in the DNA backbone. Locked nucleic acids, peptide nucleic acids, borane nucleic acids and phosphorothioate nucleic acids have been synthesised and used in assembling nanoparticles. These types of structures are of particular interest because they open up new directions in the use of DNA nanostructures in more challenging environments such as rough surfaces, presence of nucleases and low ionic strength solutions. They are also used to enhance the binding strength between building units and new reports on the possibility of coupling backbone modifications with photoswitching abilities are emerging.^250^

The field of DNA chemistry is very broad, and it has advanced greatly in recent years, enriching the library of structures that can be employed to master the self-assembly of nanoparticles. Many chemical modifications of oligonucleotides have been reported,^251^ most of which have not yet been explored in the self-assembly of nanoparticles. Of particular interest are the so-called expanded nucleoside analogues, which include fluorescent nucleosides and size-expanded DNA.^252–257^ Other examples include morpholino DNA analogues where the anionic phosphates have been replaced with uncharged phosphorodiamidate groups, eliminating ionisation in the usual pH range, and oligonucleotides with 2′-*O*-methyl or 2′-methoxyethyl modified sugars, which are not susceptible to enzymatic degradation and form stable duplexes with RNA.^258^ Many of the aforementioned oligonucleotides are commercially available, making them attractive candidates for nanoparticle related applications.^183,259,260^ A plethora of PNA analogues have been reported with improved solubility,^261^ which can produce nanoparticles with new properties. Finally, several analogues of DNA exist where the backbone is positively charged,^262^ and could potentially be used to create novel cationic nanoparticle assemblies.

DNA nanotechnology has revolutionised the field of nanoparticle assembly owning to the precision and programmability of DNA, resulting in the construction of novel materials that possess customised chemical and physical properties. The methods to control the synthesis of various types of inorganic nanoparticles have significantly advanced the last decade and nowadays a broad range of nanoparticles with various morphologies and different chemical compositions can be easily fabricated. Moreover, a new method involving complex oligonucleotides structures for the programmable digital fabrication of 3D inorganic nanostructures with prescribed shapes, dimensions, and surface modifications has emerged in recent years.^263–265^ In this strategy, a three-dimensional DNA origami nanostructure featuring an internal cavity is used as mould. A small metal nanoparticle acts as seed within the cavity, then it grows into a larger metal nanoparticle filling the entire cavity, thereby replicating its prescribed 3D shape. The remaining DNA mould additionally acts as a spatially programmable functionalisation surface. This strategy has been successfully employed not only for the synthesis of individual gold and silver nanostructures, but also for highly hierarchical modular assemblies, based on moulds with different diameters and additional docking sites and junctions.^266^ This way metal nanostructures firmly enclosed by DNA are obtained, owning controlled spacings and constraints between metal interfaces. Analogously, three-dimensional DNA patterns can be transferred also to polymeric particles. Such “printed” particles are moulded inside the DNA cage with a precise and tuneable number of repeating units and prescribed valency.^267,268^ Furthermore, progress in research related to nanoparticle-ligand interface, enables an enriched availability of building units that could be used in making new self-assembled materials.^269^

Over the past few decades, the production of dynamic DNA-nanoparticle assemblies has been realised, owning to the use of stimuli-responsive DNA.^35^ The resulting stimuli-responsive nanostructures can overcome the limitations of traditional DNA-nanoparticle assemblies enabling reversible-conformational control of the nanostructures upon external interrogation (*e.g.* light, pH, temperature, metal ions and electricity). An exciting toolkit for the construction of reversible, multi-responsive nanoparticle assemblies with versatile functionalities, could include the use of DNA nano switches,^270^ DNA walkers,^41^ DNA tweezers,^271^ and DNA Motor based functional systems in general.^272–274^ Additionally, the constant development of new, dynamic DNA chemical modifications as well as their hybrids with various polymers,^275^ and the emergence of DNA-encoded protein nanoparticle assembly,^276^ could enable the construction of new materials.^277^ Enhancing collaborations between the research communities of DNA nanotechnology and nanomaterial design, will enable the construction of various new materials that cannot otherwise be attained. For example, novel inorganic all-inorganic replicas of complex 3D DNA architectures have been developed, endowed with exceptional thermal and mechanical stability.^278,279^

While it is difficult to predict where this new exciting interdisciplinary field of research will lead, we foresee that the new materials will possess tuneable collective properties by design that will be useful in many areas of applications such as nanomedicine, sensing, catalysis, energy conversion/storage and optics to name few.

## Conflicts of interest

There are no conflicts of interest.

## Supplementary Material
